# The level of antimicrobial resistance of sewage isolates is higher than that of river isolates in different *Escherichia coli* lineages

**DOI:** 10.1038/s41598-020-75065-x

**Published:** 2020-10-21

**Authors:** Yoshitoshi Ogura, Takuya Ueda, Kei Nukazawa, Hayate Hiroki, Hui Xie, Yoko Arimizu, Tetsuya Hayashi, Yoshihiro Suzuki

**Affiliations:** 1 Division of Microbiology, Department of Infectious Medicine, Kurume University School of Medicine, Kurume, Fukuoka 830-0011 Japan; 2 Department of Bacteriology, Graduate School of Medical Sciences, Kyushu University, Fukuoka, 812-8582 Japan; 3 Department of Civil and Environmental Engineering, Faculty of Engineering, University of Miyazaki, Miyazaki, 889-2192 Japan; 4 Department of Medicine and Biosystemic Science, Graduate School of Medical Sciences, Kyushu University, Fukuoka, 812-8582 Japan

**Keywords:** Antimicrobials, Environmental microbiology

## Abstract

The dissemination of antimicrobial-resistant bacteria in environmental water is an emerging concern in medical and industrial settings. Here, we analysed the antimicrobial resistance of *Escherichia coli* isolates from river water and sewage by the use of a combined experimental phenotypic and whole-genome-based genetic approach. Among the 283 tested strains, 52 were phenotypically resistant to one or more antimicrobial agents. The *E. coli* isolates from the river and sewage samples were phylogenetically indistinguishable, and the antimicrobial-resistant strains were dispersedly distributed in a whole-genome-based phylogenetic tree. The prevalence of antimicrobial-resistant strains as well as the number of antimicrobials to which they were resistant were higher in sewage samples than in river samples. Antimicrobial resistance genes were more frequently detected in strains from sewage samples than in those from river samples. We also found that 16 river isolates that were classified as *Escherichia* cryptic clade V were susceptible to all the antimicrobials tested and were negative for antimicrobial resistance genes. Our results suggest that *E. coli* strains may acquire antimicrobial resistance genes more frequently and/or antimicrobial-resistant *E. coli* strains may have higher rates of accumulation and positive selection in sewage than in rivers, irrespective of their phylogenetic distribution.

## Introduction

The emergence and spread of antimicrobial-resistant bacteria have been globally recognized as being among the most important issues in medical and industrial fields^1^. In medical institutions, nosocomial infections caused by antimicrobial-resistant bacteria have become a serious problem^2,3^. At present, the number of deaths caused worldwide by antimicrobial-resistant bacteria is reported to be 700,000 annually, and it is estimated that the number of deaths will increase to 10 million annually by 2050^4^. The World Health Organization^5^ and the Centers for Disease Control and Prevention^6^ have deemed antimicrobial-resistant bacteria to be a serious threat worldwide.

*Escherichia coli* is a leading cause of bloodstream and urinary tract infections, and antimicrobial-resistant *E. coli* strains, including carbapenem-resistant strains, have become a major cause for concern worldwide^5,6^. Antimicrobial-resistant *E. coli* have been detected not only in humans and livestock but also in foods, sewage and rivers^7–9^. *E. coli* is excreted from the body in the faeces and has been widely used as a faecal indicator bacterium in water environments. *E. coli* spreads to inhabited areas and in nature via water systems. Numerous studies have reported the considerable prevalence of antimicrobial-resistant *E. coli* strains in water environments, especially sewage (e.g.^10–14^).

High genetic diversity exists within the *E. coli* population^15,16^. Based on this diversity, *E. coli* strains are traditionally classified into phylogroups (A, B1, B2, C, D, E, F and G) and cryptic *Escherichia* clades (clades I to V) by PCR-based phylotyping^17–19^ or into sequence types (STs) by multilocus sequence typing (MLST)^20,21^. Using these typing methods, antimicrobial-resistant *E. coli* strains from water environments have been analysed to determine the clonal structure of these strains^22–24^. Recent advances in genome sequencing technologies have enabled large-scale and high-resolution phylogenetic analyses of bacterial strains based on whole-genome sequences. In a whole-genome sequence analysis of *E. coli* isolates from wastewater treatment plants in Canada, the U.S. and Switzerland, Zhi et al. showed evidence for the clonal expansion and global dissemination of wastewater-adapted *E. coli* clones^25^. Other whole-genome-based studies have shown that wastewater *E. coli* isolates have high genomic diversity^26^ and are phylogenetically intermixed with human and livestock isolates^27^. Additionally, Gomi et al. employed whole-genome sequencing to analyse the clonal structure of *E. coli* strains from river water and revealed the contamination of surface waters by *E. coli* strains belonging to clinically important clonal groups^28^. However, no systematic whole-genome-based study has focused on the difference in the prevalence and characteristics of antimicrobial-resistant *E. coli* strains between sewage and river water.

In this study, we isolated *E. coli* strains from sewage and river water samples that were collected at approximately the same time from closely related locations in Japan. We then performed conventional antimicrobial susceptibility testing and whole-genome-based analyses, including high-resolution phylogenetic analysis and antimicrobial resistance gene and mutation identification. We also investigated the relationship between the phenotypic and genetic antimicrobial resistance profiles of these strains.

## Results and discussion

### Isolation of *E. coli* strains from river and sewage samples

**Figure 1:**
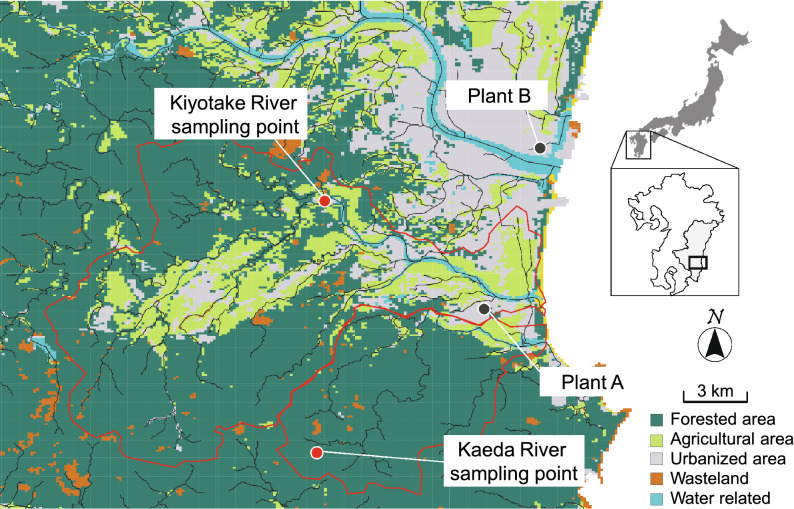
Sampling points of the Kiyotake and Kaeda Rivers in southern Japan, with the distribution of major land use classifications. The map was generated using QGIS ver.2.18.6 (https://www.qgis.org/) with the Geographic Information System (GIS) data including Land-use distribution data (https://nlftp.mlit.go.jp/ksj/gml/datalist/KsjTmplt-L03-b.html), River network data (https://nlftp.mlit.go.jp/ksj/gml/datalist/KsjTmplt-W05.html) and Watershed data (https://nlftp.mlit.go.jp/ksj/gml/datalist/KsjTmplt-W07.html).

**Figure 2:**
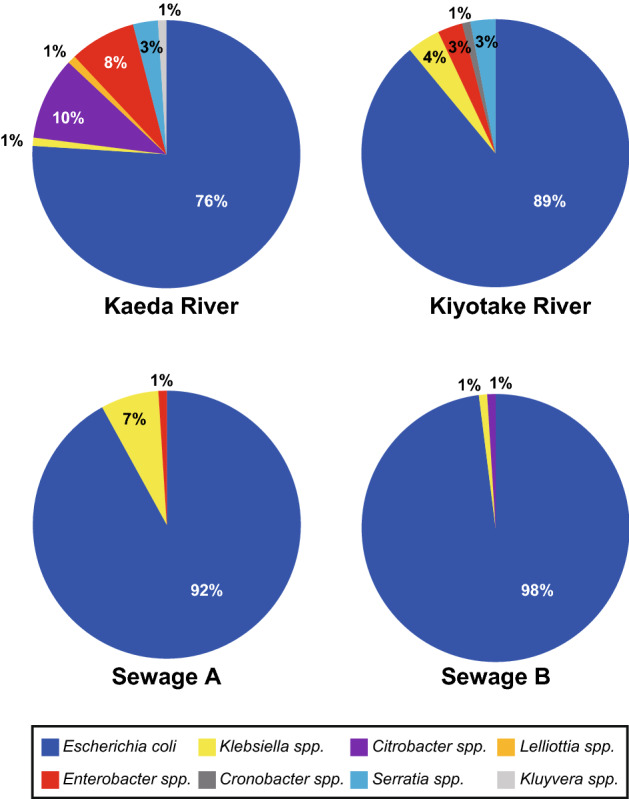
Proportions of *E. coli* and other identified species among the strains isolated from the water samples.

One water sample each was collected from two rivers (the Kaeda and Kiyotake rivers) and two sewage plants (sewage plants A and B). The Kaeda and Kiyotake rivers are in close proximity to one another in Miyazaki Prefecture, Japan; the sampling sites were located in the uppermost stream surrounded by forest and downstream of an urbanized area, respectively (Fig. 1; see “Materials and methods” for details). Sewage plants A and B are also located in Miyazaki Prefecture (Fig. 1). From each sample, 100 isolates putatively regarded as *E. coli* were obtained and then subjected to MALDI-TOF MS analysis for species confirmation. The proportion of *E. coli* and other identified bacterial species among the isolated strains is shown in Fig. 2. The 100 isolates from the Kaeda River were identified as *E. coli* (76 isolates), *Citrobacter* spp. (10 isolates), *Enterobacter* spp. (8 isolates), *Serratia* spp. (3 isolate), and other species (3 isolates). The 100 isolates from the Kiyotake River were identified as *E. coli* (89 isolates), *Klebsiella* spp. (4 isolates), *Enterobacter* spp. (3 isolates), *Serratia* spp. (3 isolates), and *Cronobacter* spp. (1 isolate). Among the 200 strains isolated from the two rivers, 17.5% were identified as other species. Ninety-two and 98 strains among the 100 strains isolated from sewage plants A and B, respectively, were identified as *E. coli*. The other major species among the sewage isolates was *Klebsiella* spp. (8 isolates).

### Phylogenetic analyses

In total, 355 strains identified as *E. coli* by MALDI-TOF MS analysis were sequenced. Among these, strains with low sequence coverage (< × 20), low completeness (≤ 99%) or a high contamination rate (≥ 5%) were excluded (n = 68). In addition, by ClermonTyping, 267 strains were classified as one of the *E. coli* phylogroups, while 16 and 4 were classified as clade V and other species*,* respectively. These 4 strains categorized as other species, which were identified as *Klebsiella pneumoniae* (n = 2), *Proteus vulgaris* (n = 1) and *Citrobacter freundii* (n = 1) by a subsequent BLAST search of the *gyrA* gene against the nr database, were excluded. Finally, 267 *E. coli* and 16 clade V strains were used in further analyses and are listed in supplementary Table S1.

**Figure 3:**
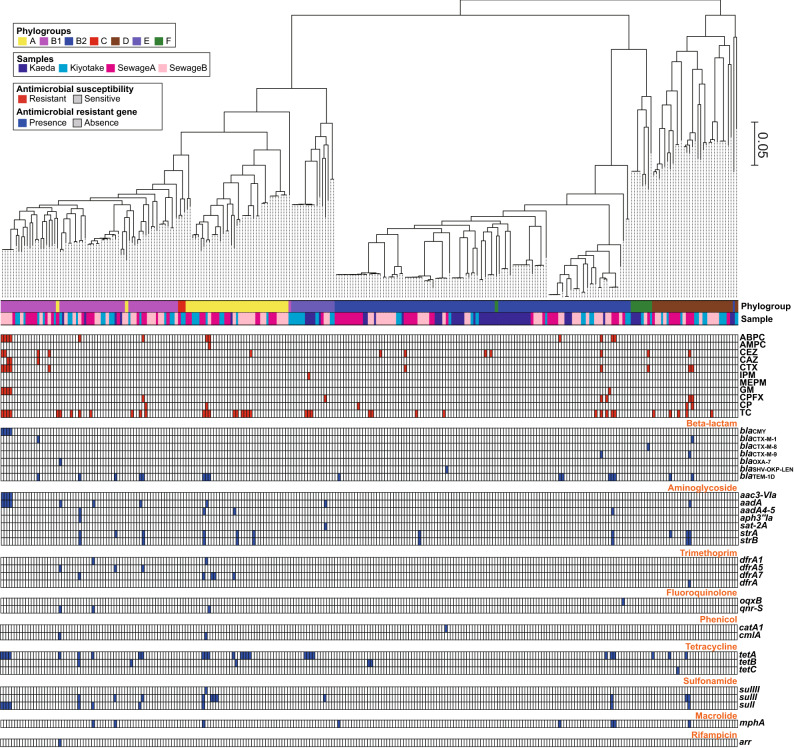
Whole-genome-based tree of 283 *E. coli* strains with phenotypic antimicrobial resistance and the presence of acquired antimicrobial resistance genes. An ML tree was constructed based on 329,863 SNP sites on 2,277 core genes. The phenotypic antimicrobial resistance and the presence of acquired antimicrobial resistance genes are indicated by red and blue, respectively.

We constructed a core gene-based maximum likelihood (ML) tree of the 267 *E. coli* strains (Fig. 3). The ML tree showed that although several subsets of strains from the same water samples were clustered together into sublineages in the tree, strains from each water sample were dispersedly distributed in the tree. The phylogroups determined by ClermonTyping mostly corresponded to the phylogenetic relationships between the strains in the ML tree, with only a few exceptions.

**Figure 4:**
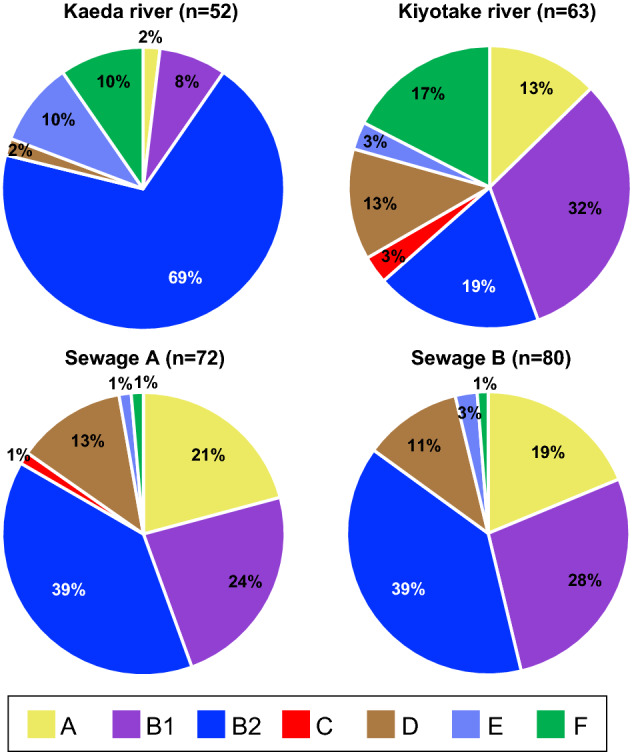
Proportions of phylogroups and clades of *E. coli* strains isolated from the water samples.

The phylogroups of the strains are summarized in Fig. 4. Among the strains isolated from the rivers, strains from the Kaeda River were mainly classified into phylogroup B2, while strains from the Kiyotake River were mainly classified into phylogroups A, B1, B2, D and F. The statistical analysis indicated that the proportions of each phylogroup were significantly different between the samples from the Kaeda and Kiyotake rivers (p < 0.05). This may be due to the difference in catchment environments, including forest and urban environments, between the rivers (Fig. 1). Regarding the sewage samples, in contrast, there was no significant difference between the samples from wastewater plants A and B in terms of the proportions of each phylogroup. Strains isolated from both sewage samples were mainly classified into phylogroups A, B1, B2 and D. Phylogroup F strains were frequently detected in both river samples but rarely detected in the sewage samples.

### Antimicrobial susceptibility

**Figure 5:**
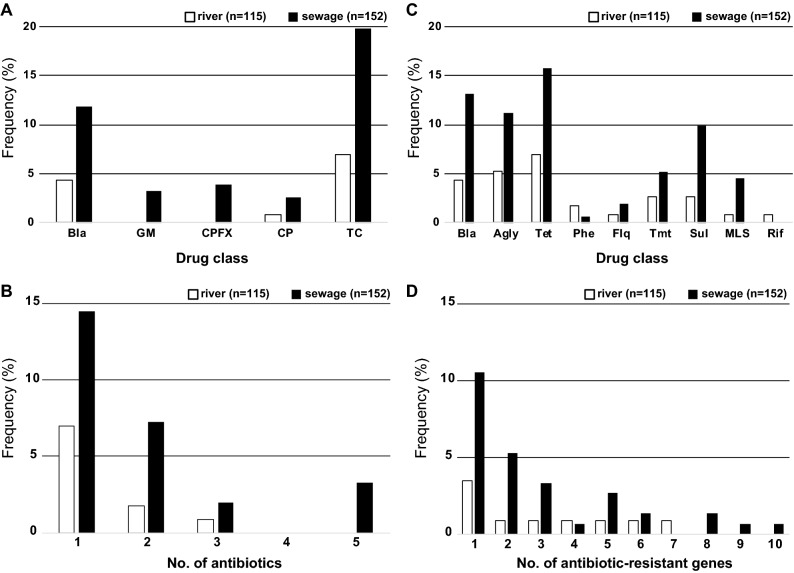
Prevalence of antimicrobial resistance phenotypes and genes in 283 *E. coli* isolates. Antimicrobial resistance profiles (**A**) and antimicrobial resistance gene content (**B**) grouped by drug class are indicated. Histograms illustrating the number of antimicrobials to which *E. coli* were phenotypically resistant (**C**) and genetically resistant (**D**) are shown.

The susceptibility of the 267 *E. coli* strains to 11 antimicrobials categorized into five classes was tested. Among these strains, 215 were susceptible to all the antimicrobials tested (ABPC, AMPC, CEZ, CAZ, CTX, IMP, MEPM, GM, CPFX, CP and TC), and 52 were resistant to one or more (up to five) antimicrobials (supplementary Table S1 and Fig. S1A). Only one resistant strain was identified for AMPC and IMP, and all strains were susceptible to MEPM. Notably, the prevalence of antimicrobial-resistant strains in sewage samples was higher than that in river samples for all antimicrobial classes (Fig. 5A). Strains resistant to multiple antimicrobials were also detected more frequently in sewage samples than in river samples (Fig. 5B).

Antimicrobial-resistant strains were detected in the river water sample that was collected from the uppermost stream of the Kaeda River, which is surrounded by forest, indicating the dissemination of antimicrobial-resistant *E. coli* to not only urban areas but also natural environments. The distribution and diffusion routes of these strains need to be clarified in future studies.

### Genetic identification of acquired antimicrobial resistance genes

The genomes of the 267 *E. coli* strains were screened for known horizontally acquired antimicrobial resistance genes. No antimicrobial resistance genes were detected in 217 strains, and one or more (up to 10) antimicrobial resistance genes were detected in 50 strains (supplementary Table S1). In total, 30 antimicrobial resistance genes classified into nine antimicrobial classes were detected in one or more (up to 27) strains. Although the distribution patterns of the antimicrobial resistance genes varied among strains from the water sample (supplementary Fig. S1B), antimicrobial resistance genes were detected more frequently in strains from sewage samples than in strains from river samples (Fig. 5C), consistent with the antimicrobial susceptibility results. In addition, strains carrying multiple antimicrobial resistance genes were also detected more frequently in the strains from sewage samples than in the strains from river samples (Fig. 5D).

It has been previously reported that a continuous influx of antimicrobial resistance genes and antimicrobial agents occurs in wastewater plants and that although the concentrations of antimicrobial agents in wastewater are relatively low, they are sufficient to exert a selective pressure, thereby increasing the prevalence of antimicrobial-resistant bacteria^29–31^. Accordingly, our results suggest that *E. coli* strains in sewage-treated water may acquire mobile genomic elements carrying antimicrobial resistance genes more frequently than those in river water and/or that resistant strains may be positively selected in sewage-treated water under selection pressures such as drug contamination.

### Phylogenetic distribution of antimicrobial-resistant strains and concordance of phenotypic and genotypic antimicrobial resistance

Both phenotypic and genotypic antimicrobial-resistant strains were dispersedly distributed in the core gene tree rather than clustered into specific sublineages (Fig. 3). This suggests that gain of acquired antimicrobial resistance genes frequently occurs in these strains. A similar phenomenon has also been reported in clinical *E. coli* strains^32,33^.

In all six CPFX-resistant strains, there were no acquired fluoroquinolone resistance genes, but at least one nonsynonymous mutation was detected in a quinolone resistance-determining region (QRDR) of the *gyrA* or *parC* gene (supplementary Fig. S2). These CPFX-resistant strains are phylogenetically diverse (Fig. 2) but share mutations in the QRDR of the *gyrA* and *parC* genes, indicating that these mutations were introduced in parallel in these strains.

All four strains carrying the acquired fluoroquinolone resistance gene were susceptible to CPFX. This is consistent with a previous report showing that the resistance determinant does not confer a high level of resistance to fluoroquinolones but rather confers reduced susceptibility to these antimicrobials^34^.

**Figure 6:**
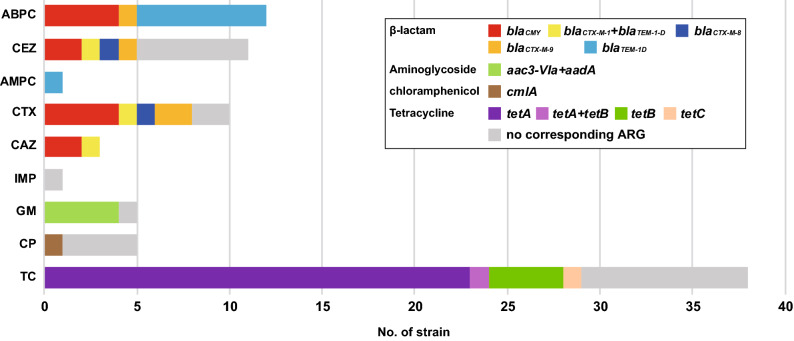
Concordance of phenotypic and genotypic antimicrobial resistance. In each drug class, the numbers of phenotypically resistant strains with or without corresponding antibiotic resistance genes (ARGs) are indicated.

For the other antimicrobials, 73% of the phenotypic resistance can be explained by the presence of known acquired antimicrobial resistance genes, but the remaining 27% cannot be explained, especially the resistance to CEZ and CP (Fig. 6 and supplementary Table S1). These strains may have other resistance mechanisms, such as drug efflux pumps and modified membrane permeability, against these antimicrobials. In a previous study of clinical enteropathogenic *E. coli* strains, more than 95% of phenotypic resistance was explained by the presence of known genetic determinants of antimicrobial resistance^32^. Environmental *E. coli* strains may acquire genetically unrecognized resistance mechanisms against antimicrobial agents more frequently than clinical *E. coli* strains.

### Carbapenem resistance

Carbapenems are β-lactam antimicrobials with a broad antimicrobial spectrum and are regarded as the last treatment option for severe infections caused by both gram-negative and gram-positive bacteria. In this study, one strain (KAEDA-061) was resistant to IMP, but no known acquired antimicrobial resistance gene for IMP was detected in this strain (Fig. 6 and supplementary Table S1). In addition to acquired antimicrobial resistance, reduced expression of porins results in decreased susceptibility to antimicrobials in bacteria^35^. In *E. coli*, OmpC, OmpF and PhoE are porins that control the influx of antimicrobials, and clinical isolates with carbapenem resistance often show loss of these porins^36,37^. We thus performed genomic comparisons of KAEDA-061 with the strains KAEDA-073 and KAEDA-068, which are phylogenetically closely related to KAEDA-061 but are IMP susceptible. Among the ten SNPs that were specifically present in strain KAEDA-061, five that were located on genes encoding a potassium transporter, an ATP phosphoribosyltransferase, a dihydropteridine reductase, a nitrate/nitrite transporter and a lipopolysaccharide (LPS) assembly outer membrane protein were nonsynonymous or nonsense mutations (supplementary Table S2). In addition, 20 genes were identified as KAEDA-061-specific genes by pangenome comparisons with strains KAEDA-073 and KAEDA-068 (supplementary Table S3). Although genes known to be related to carbapenem susceptibility were not included among these genes, the contribution of these genes and mutations to IMP resistance should be analysed in future studies.

### Antimicrobial resistance of clade V strains

Among the strains from river samples, 16 (5 and 11 in Kaeda and Kiyotake, respectively) were phylogenetically distinct from the other *E. coli* strains (supplementary Fig. S3) and classified as clade V by ClermonTyping (supplementary Table S1). Clade V strains are thought to be better adapted to the environment than *E. coli* and clade I strains^38^. It has also been reported that clade V strains exhibit low antimicrobial resistance to seven drugs (nalidixic acid, chloramphenicol, kanamycin, streptomycin, tetracycline, amoxicillin, and sulfamethoxazole). In this study, although resistant strains were distributed fairly evenly across the phylogroups, clade V strains were susceptible to all the antimicrobials tested and negative for any known acquired antimicrobial resistance genes (supplementary Fig. S4), suggesting that these strains were not under strong selection pressure from antimicrobial agents due to their low degrees of association with human and livestock populations.

## Conclusion

In this study, we isolated and analysed more than 300 *E. coli* strains from two river and two sewage samples. The whole-genome-based phylogenetic tree revealed great phylogenetic diversity among *E. coli* isolates from water samples. Even though both phenotypically and genotypically antimicrobial-resistant strains were dispersedly distributed in the whole-genome-based tree, resistant strains were detected more frequently in the sewage samples than in the river samples. Although 27% of phenotypic resistance could not be explained by the presence of known acquired antimicrobial resistance genes, 73% could be. Sixteen river isolates that were classified as clade V were susceptible to all the antimicrobials tested and negative for any known acquired antimicrobial resistance genes, suggesting low degrees of association with human and livestock populations. Although our study was not performed globally or seasonally, the present results imply that antimicrobial-resistant *E. coli* belonging to various phylogenetic lineages accumulate, emerge and/or are selected more frequently in sewage than in natural rivers.

## Materials and methods

### Sampling

Samples of river water were collected from the Kaeda (length of river channel 17.5 km, catchment area 53.8 km^2^) and Kiyotake (28.8 km, 166.4 km^2^) rivers, which vary in catchment environments, in Miyazaki, Japan (Fig. 1). The sampling site of the Kaeda River was located in the uppermost stream of the river, which is surrounded by forest and where there was no anthropogenic activity. Therefore, the sample from the Kaeda River was considered to be natural water. In contrast, the site of the Kiyotake River was downstream of an urbanized area (population of 12,000), so the sample from the Kiyotake River was considered to be urban water. The samples were collected from the Kaeda and Kiyotake rivers in October and November 2017, respectively. Sewage samples were collected from an influent ditch before primary settling at plants A and B. Plant A treats sewage from a region with a population of approximately 10,000 people, and its average daily flow volume is 6300 m^3^. Plant B treats sewage from a region with a population of approximately 163,000 people, and its average daily flow volume is 94,000 m^3^. The samples were collected from plants A and B in August 2017 and October 2017, respectively. The collected water samples were stored in sterile 1-L polyethylene bottles and immediately transported for microbial analysis. Microbial analysis was initiated within 4 h of sampling.

### Isolation and identification of *E. coli*

Isolation of *E. coli* from water samples was described previously^39^. In brief, samples were filtered through a membrane filter (0.45-µm pore, 47-mm diameter, sterile, mixed cellulose ester; Advantec, Tokyo, Japan) and incubated on CHROMagar ECC plates (CHROMagar, Paris, France) at 37 °C for 24 h. After incubation, 100 single mauve colonies putatively identified as *E. coli* were picked from the ECC agar plates and purified by repeated single-colony isolation on the same medium. The isolates were incubated on brain heart infusion (BHI) agar plates (1.5% agar; Becton, Dickinson and Company, NJ, USA) at 37 °C for 18 h and subjected to species identification.

Then, matrix-assisted laser desorption/ionization-time of flight mass spectrometry (MALDI-TOF MS) analysis was used for species identification^39^. An aliquot (1.0 μL) of colony suspension was spotted directly onto a 384-well stainless-steel target plate (MTP 384, Bruker Daltonics, Billerica, MA, USA). Following air-drying for 10 min, a template was overlaid with 1.0 μL of the matrix solution. All samples were analysed using an Autoflex III TOF/TOF (Bruker Daltonics, Billerica, MA, USA) operated in the linear positive mode within a mass range of 2000–20,000 Da, according to the manufacturer's instructions. For database construction and validation, measurements were performed in the auto-execute mode using Flex Control 3.4 software (Bruker Daltonics). The software settings were as follows: linear positive, 3–20 kDa; detector gain, 2691 V; laser shots, 40–200; laser power, 30%. A Bruker bacterial test standard (part no. 8255343, Bruker Daltonics) was used for instrument calibration. Recorded mass spectra were analysed with the MALDI Biotyper Compass (Bruker Daltonics) under standard settings. The MALDI Biotyper output is a log score value in the range of 0.000 to 3.000. The *E. coli* identification score was greater than 2.000.

### Antimicrobial susceptibility testing

The minimum inhibitory concentration (MIC) of each antimicrobial agent was determined by the agar dilution method according to the Clinical and Laboratory Standards Institute (CLSI) guidelines^40^. The *E. coli* isolates were cultured at 37 °C for 18 h in Mueller–Hinton broth (Becton Dickinson, Sparks, MD, USA) and then diluted to a final concentration corresponding to the 0.5 McFarland turbidity standard with fresh Mueller–Hinton broth. Inocula were then applied to the surface of Mueller–Hinton agar (1.5% agar) plates containing graded concentrations of each antimicrobial in a microplate (Sakuma Co., Tokyo, Japan). The plates were incubated at 37 °C for 18 h, and MICs were determined. MIC breakpoints for resistance were based on the CLSI criteria.

The antimicrobials used in the current study included ampicillin (ABPC, graded concentrations from 4–64 µg/mL) (Wako Pure Chemical Industries, Ltd., Osaka, Japan) and amoxicillin (AMPC, 4–64 µg/mL) (Wako Pure Chemical Industries) as representative penicillins; imipenem (IMP, 0.5–8 µg/mL) (Wako Pure Chemical Industries) and meropenem (MEPM, 4–64 µg/mL) (Wako Pure Chemical Industries) as representative carbapenems; cefazolin (CEZ, 1–16 µg/mL), cefotaxime (CTX, 0.5–8 µg/mL) (Wako Pure Chemical Industries) and ceftazidime (CAZ, 2–32 µg/mL) (Wako Pure Chemical Industries) as representative cephem antimicrobials; gentamycin (GM, 2–32 µg/mL) (Wako Pure Chemical Industries) as a representative aminoglycoside; ciprofloxacin (CPFX, 0.5–8 µg/mL) (Wako Pure Chemical Industries) as a representative fluoroquinolone; chloramphenicol (CP, 4–64 µg/mL) (Sigma-Aldrich) as a representative phenicol; and tetracycline (TC, 2–32 µg/mL) (Wako Pure Chemical Industries) as a representative tetracycline. *E. coli* strain ATCC 25,922 was used as a reference.

### Genome sequencing, assembly and annotation

Genomic DNA was purified from 1 mL of an overnight culture of each strain using the DNeasy Blood and Tissue Kit (Qiagen, Valencia, CA, USA). Genomic DNA libraries were prepared using the NEBNext Ultra II DNA Library Prep Kit (New England Biolabs, Beverly, MA) and sequenced using the Illumina HiSeq 2500 platform (Illumina, San Diego, CA, USA) to generate 150-bp paired-end reads. Genome assembly, scaffolding, and gap-closing of the Illumina sequence reads were performed using the Platanus assembler^41^. Strains with low sequence coverage (< × 20) were excluded from the analyses. The completeness and potential contamination of the assembled genomes were calculated using CheckM software^42^. Only genomes with greater than 99% completeness and less than 5% contamination were used in further analyses. Annotation was carried out with the DDBJ Fast Annotation and Submission Tool (DFAST)^43^. All sequence data generated in this study have been submitted to the NCBI BioProject database (BioProject; https://www.ncbi.nlm.nih.gov/bioproject/) under accession number PRJDB9692.

### Phylogroup determination

Phylogroups were determined by ClermonTyping^44^ using the assembled draft genome sequences with the default parameters.

### Core gene-based phylogenetic analysis

Core genes were identified using Roary^45^ (≥ 80% nucleotide sequence identity). Single nucleotide polymorphism (SNP) sites were extracted from the core gene alignment using snp-sites^46^. ML phylogenetic trees were constructed using RAxML^47^ from the concatenated SNP alignment with the GTR-GAMMA model of nucleotide substitution with 100 bootstrap replicates. The ML phylogenetic trees were visualized and annotated using iTOL^48^.

### Detection of acquired antimicrobial resistance genes

The detection of acquired antimicrobial resistance genes was performed by a read mapping-based strategy using the SRST2 program^49^ and the database file ARGannot_r3.fasta with the default setting.

### Statistical analysis

Chi-squared tests were performed to compare the relative abundances of the phylogroups between the river and sewage samples. Likewise, differences in the relative abundances of antimicrobial-resistant *E. coli* between the river and sewage samples were analysed. All statistical analyses were performed using SPSS 23.0 for Windows (IBM SPSS Statistics).

## Supplementary information

Supplementary Information.
