# Supplementary material for: The level of antimicrobial resistance of sewage isolates is higher than that of river isolates in different Escherichia coli lineages
Source: Sci Rep. 2020 Oct 21;10:17880. doi: 10.1038/s41598-020-75065-x (PMC7578040; doi:10.1038/s41598-020-75065-x)
Supplement: Supplementary file 1 — Supplementary Information. [file 41598_2020_75065_MOESM1_ESM.pdf]

Supporting Information for **'The level of antimicrobial resistance of sewage isolates is higher than that of river isolates in different *Escherichia coli* lineages'**

Yoshitoshi Ogura[1,2*], Takuya Ueda[3], Kei Nukazawa[3], Hayate Hiroki[3], Hui Xie[3], Yoko Arimizu[2,4], Tetsuya Hayashi[2] and Yoshihiro Suzuki[3*]

[1]Division of Microbiology, Department of Infectious Medicine, Kurume University School of Medicine, Kurume, Fukuoka 830-0011, Japan; [2]Department of Bacteriology, Graduate School of Medical Sciences, Kyushu University, Fukuoka 812-8582, Japan; [3]Department of Civil and Environmental Engineering, Faculty of Engineering, University of Miyazaki, Miyazaki 889-2192, Japan; [4]Department of Medicine and Biosystemic Science, Graduate School of Medical Sciences, Kyushu University, Fukuoka 812-8582, Japan.

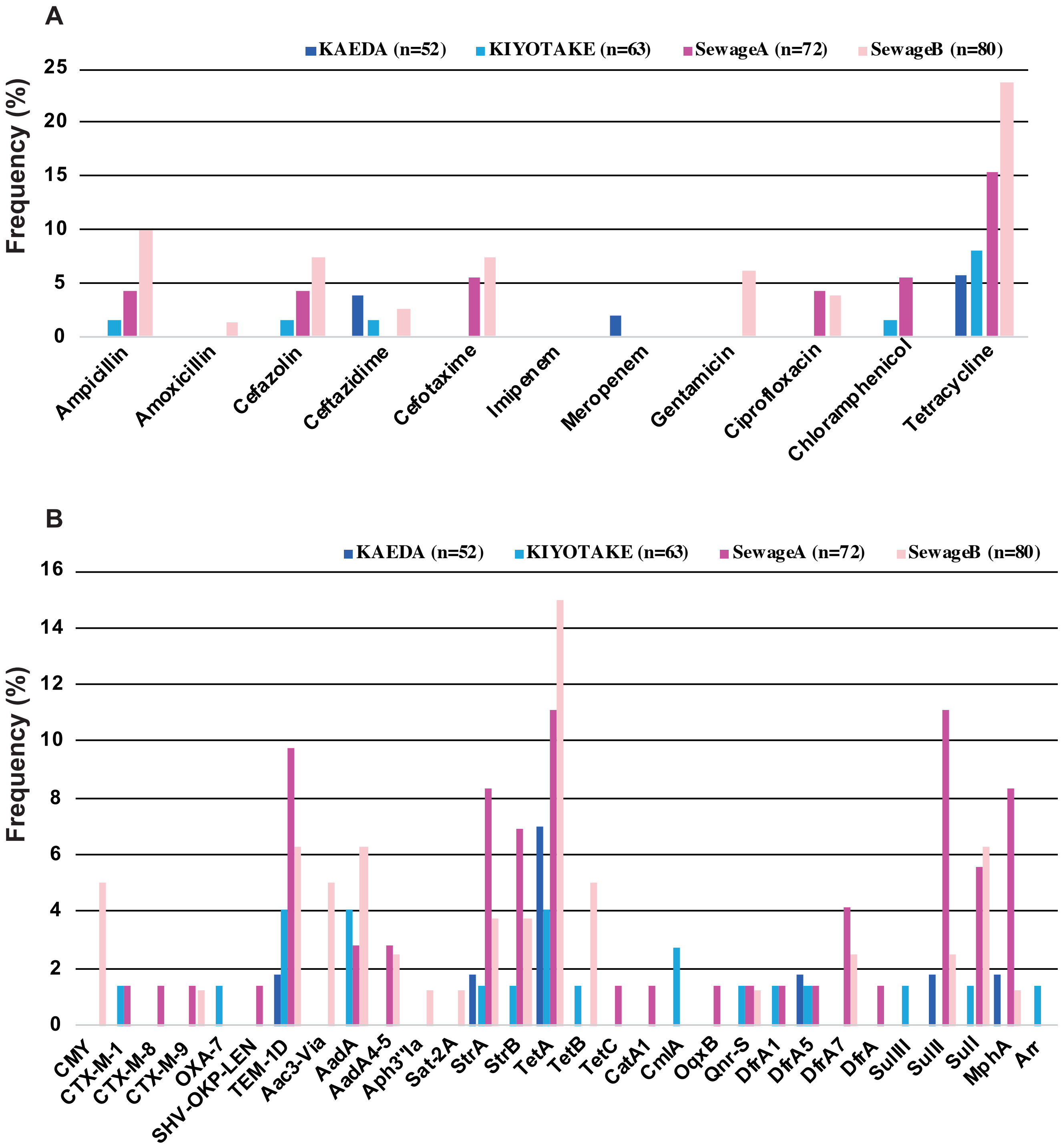

Supplemental Figure S1. Prevalence of antibiotic resistance phenotypes (A) and antibiotic resistance gene content (B) in *E. coli* isolates from rivers and sewage.

A. *gyrA*

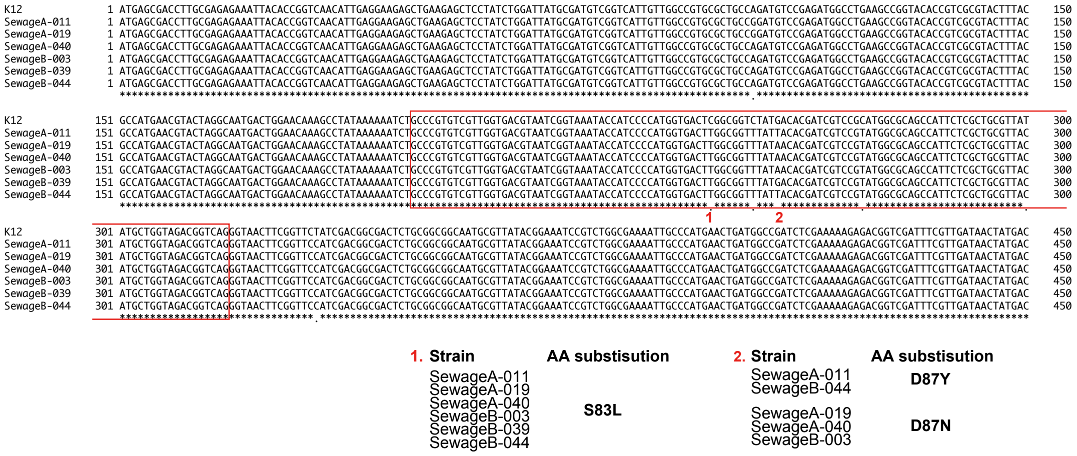

B. *parC*

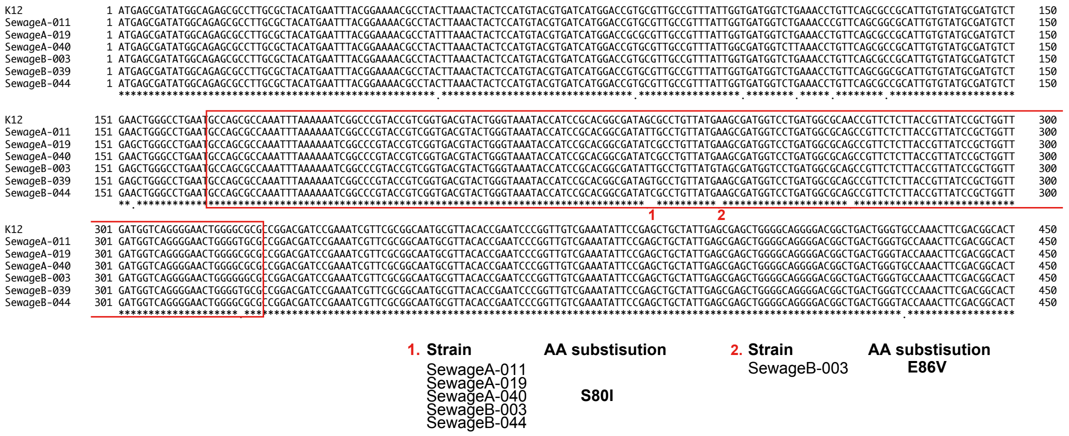

Supplemental Figure S2. Nucleotide sequence alignments of the N-terminal region of the *gyrA* and *parC* genes of quinolone-resistant strains.

E. coli strain K-12 MG1655 was included as a reference. The quinolone resistance–determining region (QRDR) is enclosed by a red line. Nonsynonymous mutations within the QRDR are indicated by red numbers, and the corresponding strains and amino acid substitutions of each mutation are shown under the alignments.

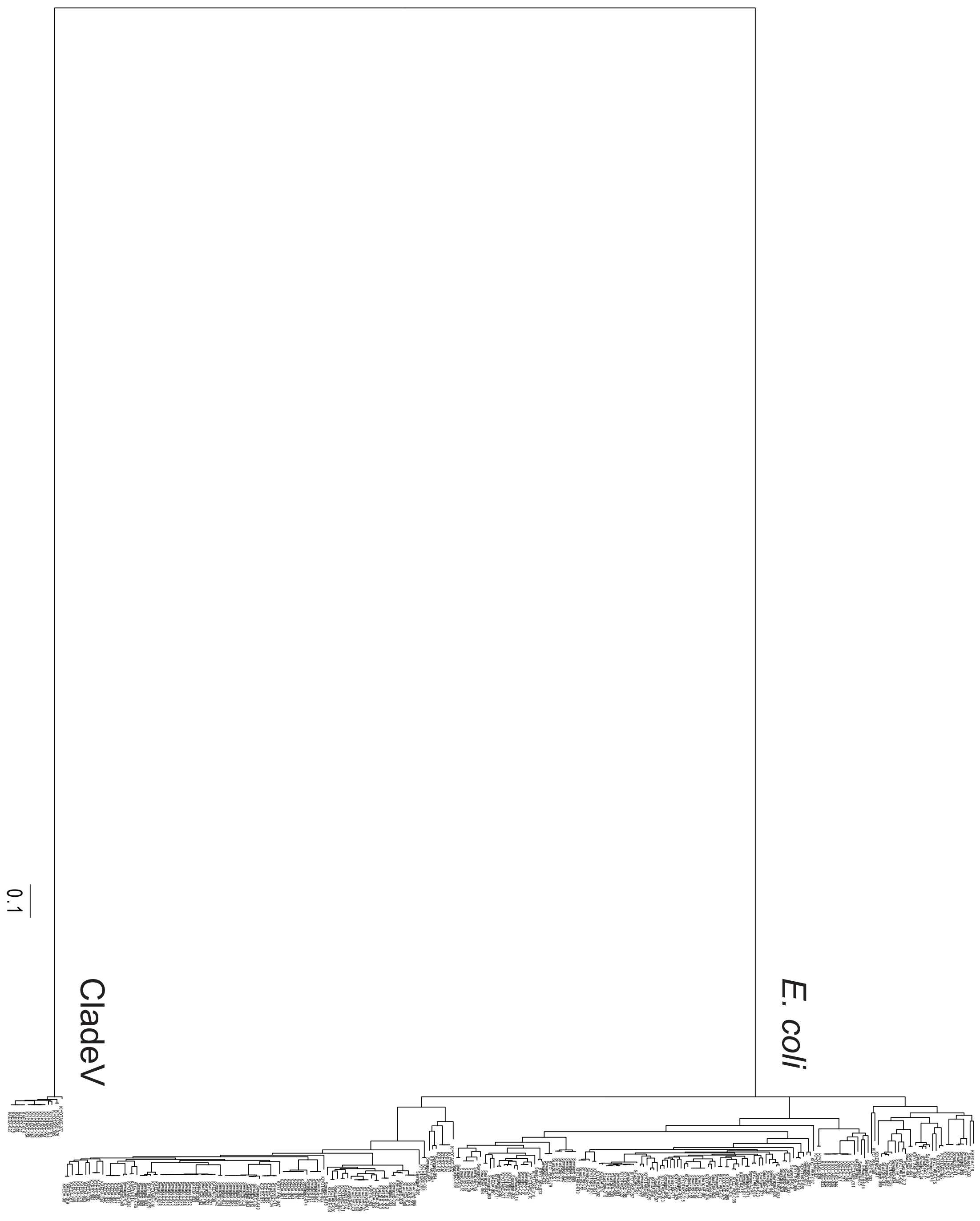

Supplemental Figure S3. Core gene-based maximum likelihood (ML) tree of 267 *E. coli* and 16 clade V strains.

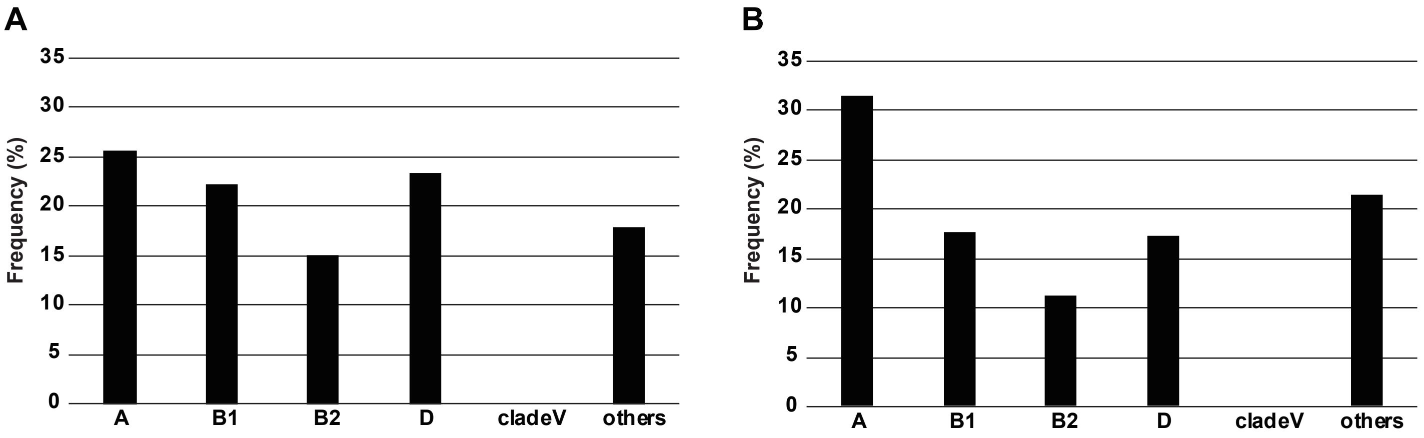

Supplemental Figure S4. Distribution of phenotypically and genotypically resistant strains across the phylogroups and clade.

Supplemental table S1. Characteristics of *E. coli* strains analyzed in this study

Strain	Phylogroup	Statistics of genome assembly						Antimicrobial suseptibility											Detection of antibiotic resistance genes (0: absent, 1: present)																													
		No. of reads	Total read length	average coverage	No. of scaffolds	longest	total scaffold length	ABPC	AMPC	CEZ	CAZ	CTX	IPM	MEPM	GM	CPFX	CP	TC	CMY_Bla	CTX-M-1_Bla	CTX-M-8_Bla	CTX-M-9_Bla	OXA-7_Bla	SHV-OKP-LEN_Bla	TEM-1D_Bla	Aac3-VIa_AGly	AadA_AGly	AadA4-5_AGly	Aph3''Ia_AGly	Sat-2A_AGly	StrA_AGly	StrB_AGly	TetA_Tet	TetB_Tet	TetC_Tet	CatA1_Phe	CmlA_Phe	OqxB_Flq	Qnr-S_Flq	DfrA1_Tmt	DfrA5_Tmt	DfrA7_Tmt	DfrA_Tmt	SulIII_Sul	SulII_Sul	SulI_Sul	MphA_MLS	Arr_Rif
KAEDA-002	B2	1,449,362	218,853,662	35	38	1132464	4774269	S	S	S	S	S	S	S	S	S	S	S	0	0	0	0	0	0	0	0	0	0	0	0	0	0	0	0	0	0	0	0	0	0	0	0	0	0	0	0	0	0
KAEDA-003	B2	1,472,378	222,329,078	32	106	505076	5130873	S	S	S	S	S	S	S	S	S	S	S	0	0	0	0	0	0	0	0	0	0	0	0	0	0	0	0	0	0	0	0	0	0	0	0	0	0	0	0	0	0
KAEDA-004	B2	1,612,778	243,529,478	39	160	460175	5101025	S	S	S	S	S	S	S	S	S	S	S	0	0	0	0	0	0	0	0	0	0	0	0	0	0	0	0	0	0	0	0	0	0	0	0	0	0	0	0	0	0
KAEDA-006	B2	1,669,628	252,113,828	49	28	774686	4789107	S	S	S	S	S	S	S	S	S	S	S	0	0	0	0	0	0	0	0	0	0	0	0	0	0	0	0	0	0	0	0	0	0	0	0	0	0	0	0	0	0
KAEDA-007	B2	1,635,854	247,013,954	44	81	881688	5181669	S	S	S	S	S	S	S	S	S	S	S	0	0	0	0	0	0	0	0	0	0	0	0	0	0	0	0	0	0	0	0	0	0	0	0	0	0	0	0	0	0
KAEDA-008	cladeV	1,451,560	219,185,560	35	184	325190	4937018	S	S	S	S	S	S	S	S	S	S	S	0	0	0	0	0	0	0	0	0	0	0	0	0	0	0	0	0	0	0	0	0	0	0	0	0	0	0	0	0	0
KAEDA-013	F	1,131,860	170,910,860	30	69	711131	5186584	S	S	S	S	S	S	S	S	S	S	S	0	0	0	0	0	0	0	0	0	0	0	0	0	0	0	0	0	0	0	0	0	0	0	0	0	0	0	0	0	0
KAEDA-014	B2	1,182,618	178,575,318	35	25	1129148	4664489	S	S	S	S	S	S	S	S	S	S	S	0	0	0	0	0	0	0	0	0	0	0	0	0	0	0	0	0	0	0	0	0	0	0	0	0	0	0	0	0	0
KAEDA-016	E	1,423,064	214,882,664	39	79	688234	5140090	S	S	S	S	S	S	S	S	S	S	S	0	0	0	0	0	0	0	0	0	0	0	0	0	0	1	0	0	0	0	0	0	0	0	0	0	0	0	0	0	0
KAEDA-017	B2	1,569,840	237,045,840	47	25	774660	4791005	S	S	S	S	S	S	S	S	S	S	S	0	0	0	0	0	0	0	0	0	0	0	0	0	0	0	0	0	0	0	0	0	0	0	0	0	0	0	0	0	0
KAEDA-018	D	1,245,290	188,038,790	35	84	562538	5130245	S	S	S	S	S	S	S	S	S	S	S	0	0	0	0	0	0	0	0	0	0	0	0	0	0	0	0	0	0	0	0	0	0	0	0	0	0	0	0	0	0
KAEDA-019	B2	1,294,858	195,523,558	36	103	708249	5135314	S	S	S	S	S	S	S	S	S	S	S	0	0	0	0	0	0	0	0	0	0	0	0	0	0	0	0	0	0	0	0	0	0	0	0	0	0	0	0	0	0
KAEDA-022	cladeV	1,425,096	215,189,496	41	156	325299	4939692	S	S	S	S	S	S	S	S	S	S	S	0	0	0	0	0	0	0	0	0	0	0	0	0	0	0	0	0	0	0	0	0	0	0	0	0	0	0	0	0	0
KAEDA-025	cladeV	1,634,146	246,756,046	47	142	452936	4938444	S	S	S	S	S	S	S	S	S	S	S	0	0	0	0	0	0	0	0	0	0	0	0	0	0	0	0	0	0	0	0	0	0	0	0	0	0	0	0	0	0
KAEDA-031	B2	1,443,586	217,981,486	39	73	882242	5174258	S	S	S	S	S	S	S	S	S	S	S	0	0	0	0	0	0	0	0	0	0	0	0	0	0	0	0	0	0	0	0	0	0	0	0	0	0	0	0	0	0
KAEDA-032	B2	1,481,736	223,742,136	44	29	774831	4790079	S	S	S	S	S	S	S	S	S	S	S	0	0	0	0	0	0	0	0	0	0	0	0	0	0	0	0	0	0	0	0	0	0	0	0	0	0	0	0	0	0
KAEDA-033	B2	1,413,858	213,492,558	31	70	576347	5046063	S	S	S	S	S	S	S	S	S	S	S	0	0	0	0	0	0	0	0	0	0	0	0	0	0	0	0	0	0	0	0	0	0	0	0	0	0	0	0	0	0
KAEDA-034	B2	1,354,508	204,530,708	35	72	439191	4984748	S	S	S	S	S	S	S	S	S	S	S	0	0	0	0	0	0	0	0	0	0	0	0	0	0	0	0	0	0	0	0	0	0	0	0	0	0	0	0	0	0
KAEDA-035	cladeV	1,271,344	191,972,944	31	158	452850	4934262	S	S	S	S	S	S	S	S	S	S	S	0	0	0	0	0	0	0	0	0	0	0	0	0	0	0	0	0	0	0	0	0	0	0	0	0	0	0	0	0	0
KAEDA-037	B2	1,337,656	201,986,056	36	81	882188	5173162	S	S	S	S	S	S	S	S	S	S	S	0	0	0	0	0	0	0	0	0	0	0	0	0	0	0	0	0	0	0	0	0	0	0	0	0	0	0	0	0	0
KAEDA-038	B2	1,381,810	208,653,310	39	27	930532	4964587	S	S	S	S	S	S	S	S	S	S	S	0	0	0	0	0	0	0	0	0	0	0	0	0	0	0	0	0	0	0	0	0	0	0	0	0	0	0	0	0	0
KAEDA-039	B2	1,205,166	181,980,066	36	22	796613	4724417	S	S	S	S	S	S	S	S	S	S	S	0	0	0	0	0	0	0	0	0	0	0	0	0	0	0	0	0	0	0	0	0	0	0	0	0	0	0	0	0	0
KAEDA-040	B2	1,253,410	189,264,910	37	27	774862	4789680	S	S	S	S	S	S	S	S	S	S	S	0	0	0	0	0	0	0	0	0	0	0	0	0	0	0	0	0	0	0	0	0	0	0	0	0	0	0	0	0	0
KAEDA-044	B2	1,311,626	198,055,526	39	38	661813	4817495	S	S	R	S	S	S	S	S	S	S	S	0	0	0	0	0	0	0	0	0	0	0	0	0	0	0	0	0	0	0	0	0	0	0	0	0	0	0	0	0	0
KAEDA-046	F	1,298,774	196,114,874	35	65	574382	5184382	S	S	S	S	S	S	S	S	S	S	S	0	0	0	0	0	0	0	0	0	0	0	0	0	0	0	0	0	0	0	0	0	0	0	0	0	0	0	0	0	0
KAEDA-047	B2	1,442,162	217,766,462	42	22	1489227	4728633	S	S	S	S	S	S	S	S	S	S	S	0	0	0	0	0	0	0	0	0	0	0	0	0	0	0	0	0	0	0	0	0	0	0	0	0	0	0	0	0	0
KAEDA-048	B2	1,329,962	200,824,262	33	46	661364	4815004	S	S	S	S	S	S	S	S	S	S	S	0	0	0	0	0	0	0	0	0	0	0	0	0	0	0	0	0	0	0	0	0	0	0	0	0	0	0	0	0	0
KAEDA-049	B2	2,259,698	341,214,398	49	158	803651	5539709	S	S	S	S	S	S	S	S	S	S	S	0	0	0	0	0	0	0	0	0	0	0	0	0	0	0	0	0	0	0	0	0	0	0	0	0	0	0	0	0	0
KAEDA-052	B2	1,206,222	182,139,522	32	102	752762	5063812	S	S	S	S	S	S	S	S	S	S	S	0	0	0	0	0	0	0	0	0	0	0	0	0	0	0	0	0	0	0	0	0	0	0	0	0	0	0	0	0	0
KAEDA-053	B2	1,191,312	179,888,112	33	58	944145	4952697	S	S	S	S	S	S	S	S	S	S	S	0	0	0	0	0	0	0	0	0	0	0	0	0	0	0	0	0	0	0	0	0	0	0	0	0	0	0	0	0	0
KAEDA-054	B1	1,493,714	225,550,814	46	48	1188233	4629017	S	S	S	S	S	S	S	S	S	S	S	0	0	0	0	0	0	0	0	0	0	0	0	0	0	0	0	0	0	0	0	0	0	0	0	0	0	0	0	0	0
KAEDA-055	B1	1,279,590	193,218,090	36	54	758798	4887242	S	S	S	S	S	S	S	S	S	S	S	0	0	0	0	0	0	0	0	0	0	0	0	0	0	0	0	0	0	0	0	0	0	0	0	0	0	0	0	0	0
KAEDA-057	B2	1,271,186	191,949,086	38	26	774782	4791246	S	S	S	S	S	S	S	S	S	S	S	0	0	0	0	0	0	0	0	0	0	0	0	0	0	0	0	0	0	0	0	0	0	0	0	0	0	0	0	0	0
KAEDA-058	B2	1,284,072	193,894,872	33	135	803449	5545418	S	S	S	S	S	S	S	S	S	S	S	0	0	0	0	0	0	0	0	0	0	0	0	0	0	0	0	0	0	0	0	0	0	0	0	0	0	0	0	0	0
KAEDA-061	E	1,182,202	178,512,502	29	159	726173	5198240	S	S	S	S	S	R	S	S	S	S	R	0	0	0	0	0	0	0	0	0	0	0	0	0	0	1	0	0	0	0	0	0	0	0	0	0	0	0	0	0	0
KAEDA-067	B2	1,227,436	185,342,836	31	304	426642	5658626	S	S	R	S	S	S	S	S	S	S	S	0	0	0	0	0	0	0	0	0	0	0	0	0	0	0	0	0	0	0	0	0	0	0	0	0	0	0	0	0	0
KAEDA-068	E	1,407,332	212,507,132	37	105	726165	5153383	S	S	S	S	S	S	S	S	S	S	R	0	0	0	0	0	0	0	0	0	0	0	0	0	0	1	0	0	0	0	0	0	0	0	0	0	0	0	0	0	0
KAEDA-070	B2	1,290,310	194,836,810	37	52	661325	4820697	S	S	S	S	S	S	S	S	S	S	S	0	0	0	0	0	0	0	0	0	0	0	0	0	0	0	0	0	0	0	0	0	0	0	0	0	0	0	0	0	0
KAEDA-072	B2	1,189,118	179,556,818	26	186	803403	5538516	S	S	S	S	S	S	S	S	S	S	S	0	0	0	0	0	0	0	0	0	0	0	0	0	0	0	0	0	0	0	0	0	0	0	0	0	0	0	0	0	0
KAEDA-073	E	1,376,746	207,888,646	34	131	726375	5174522	S	S	S	S	S	S	S	S	S	S	R	0	0	0	0	0	0	0	0	0	0	0	0	0	0	1	0	0	0	0	0	0	0	0	0	0	0	0	0	0	0
KAEDA-074	B2	1,305,708	197,161,908	32	129	882316	5165772	S	S	S	S	S	S	S	S	S	S	S	0	0	0	0	0	0	0	0	0	0	0	0	0	0	0	0	0	0	0	0	0	0	0	0	0	0	0	0	0	0
KAEDA-075	B1	1,398,826	211,222,726	36	160	310415	5095875	S	S	S	S	S	S	S	S	S	S	S	0	0	0	0	0	0	1	0	0	0	0	0	1	0	0	0	0	0	0	0	0	0	1	0	0	0	1	0	1	0
KAEDA-078	B2	1,380,826	208,504,726	38	46	1176017	4879089	S	S	S	S	S	S	S	S	S	S	S	0	0	0	0	0	0	0	0	0	0	0	0	0	0	0	0	0	0	0	0	0	0	0	0	0	0	0	0	0	0
KAEDA-079	B2	1,434,746	216,646,646	37	106	460193	5171418	S	S	S	S	S	S	S	S	S	S	S	0	0	0	0	0	0	0	0	0	0	0	0	0	0	0	0	0	0	0	0	0	0	0	0	0	0	0	0	0	0
KAEDA-081	B2	1,146,862	173,176,162	30	66	749861	4835799	S	S	S	S	S	S	S	S	S	S	S	0	0	0	0	0	0	0	0	0	0	0	0	0	0	0	0	0	0	0	0	0	0	0	0	0	0	0	0	0	0
KAEDA-083	F	1,490,834	225,115,934	42	108	487034	4833385	S	S	S	S	S	S	S	S	S	S	S	0	0	0	0	0	0	0	0	0	0	0	0	0	0	0	0	0	0	0	0	0	0	0	0	0	0	0	0	0	0
KAEDA-084	B2	1,333,504	201,359,104	37	75	882256	5173203	S	S	S	S	S	S	S	S	S	S	S	0	0	0	0	0	0	0	0	0	0	0	0	0	0	0	0	0	0	0	0	0	0	0	0	0	0	0	0	0	0
KAEDA-086	B1	1,340,170	202,365,670	37	54	738354	4731301	S	S	S	S	S	S	S	S	S	S	S	0	0	0	0	0	0	0	0	0	0	0	0	0	0	0	0	0	0	0	0	0	0	0	0	0	0	0	0	0	0
KAEDA-088	cladeV	1,329,750	200,792,250	38	167	306502	4938088	S	S	S	S	S	S	S	S	S	S	S	0	0	0	0	0	0	0	0	0	0	0	0	0	0	0	0	0	0	0	0	0	0	0	0	0	0	0	0	0	0
KAEDA-089	F	1,527,656	230,676,056	42	130	711069	5212576	S	S	S	S	S	S	S	S	S	S	S	0	0	0	0	0	0	0	0	0	0	0	0	0	0	0	0	0	0	0	0	0	0	0	0	0	0	0	0	0	0
KAEDA-090	B2	1,458,368	220,213,568	43	55	809111	4911423	S	S	S	S	S	S	S	S	S	S	S	0	0	0	0	0	0	0	0	0	0	0	0	0	0	0	0	0	0	0	0	0	0	0	0	0	0	0	0	0	0
KAEDA-091	A	1,426,484	215,399,084	44	57	713592	4691463	S	S	S	S	S	S	S	S	S	S	S	0	0	0	0	0	0	0	0	0	0	0	0	0	0	0	0	0	0	0	0	0	0	0	0	0	0	0	0	0	0
KAEDA-092	B2	1,615,684	243,968,284	42	70	522184	4792447	S	S	S	S	S	S	S	S	S	S	S	0	0	0	0	0	0	0	0	0	0	0	0	0	0	0	0	0	0	0	0	0	0	0	0	0	0	0	0	0	0
KAEDA-094	E	1,431,000	216,081,000	33	169	840466	5465806	S	S	S	S	S	S	S	S	S	S	S	0	0	0	0	0	0	0	0	0	0	0	0	0	0	0	0	0	0	0	0	0	0	0	0	0	0	0	0	0	0
KAEDA-095	B2	1,222,806	184,643,706	31	86	522330	4780286	S	S	S	S	S	S	S	S	S	S	S	0	0	0	0	0	0	0	0	0	0	0	0	0	0	0	0	0	0	0	0	0	0	0	0	0	0	0	0	0	0
KAEDA-097	F	1,477,480	223,099,480	40	156	711286	5226065	S	S	S	S	S	S	S	S	S	S	S	0	0	0	0	0	0	0	0	0	0	0	0	0	0	0	0	0	0	0	0	0	0	0	0	0	0	0	0	0	0
KAEDA-098	B2	1,424,366	215,079,266	39	55	885809	5056223	S	S	S	S	S	S	S	S	S	S	S	0	0	0	0	0	0	0	0	0	0	0	0	0	0	0	0	0	0	0	0	0	0	0	0	0	0	0	0	0	0
KIYOTAKE-001	B1	1,232,480	186,104,480	37	56	457960	4656677	S	S	S	S	S	S	S	S	S	S	S	0	0	0	0	0	0	0	0	0	0	0	0	0	0	0	0	0	0	0	0	0	0	0	0	0	0	0	0	0	0
KIYOTAKE-002	B1	1,177,580	177,814,580	28	164	337794	5037302	S	S	S	S	S	S	S	S	S	S	S	0	0	0	0	0	0	0	0	0	0	0	0	0	0	0	0	0	0	0	0	0	0	0	0	0	0	0	0	0	0
KIYOTAKE-004	B2	1,282,520	193,660,520	32	133	585856	4927719	S	S	S	S	S	S	S	S	S	S	S	0	0	0	0	0	0	0	0	0	0	0	0	0	0	0	0	0	0	0	0	0	0	0	0	0	0	0	0	0	0
KIYOTAKE-009	cladeV	1,417,208	213,998,408	38	83	360594	4669320	S	S	S	S	S	S	S	S	S	S	S	0	0	0	0	0	0	0	0	0	0	0	0	0	0	0	0	0	0	0	0	0	0	0	0	0	0	0	0	0	0
KIYOTAKE-010	A	1,357,598	204,997,298	36	124	399486	4622687	S	S	S	S	S	S	S	S	S	S	S	0	0	0	0	0	0	0	0	0	0	0	0	0	0	0	0	0	0	0	0	0	0	0	0	0	0	0	0	0	0
KIYOTAKE-011	A	1,365,174	206,141,274	37	136	205399	4859836	R	S	S	S	S	S	S	S	S	R	R	0	0	0	0	0	0	1	0	1	0	0	0	0	0	1	0	0	0	1	0	0	1	0	0	0	1	0	0	0	0
KIYOTAKE-012	A	1,407,052	212,464,852	31	83	328946	4671866	S	S	S	S	S	S	S	S	S	S	S	0	0	0	0	0	0	0	0	0	0	0	0	0	0	0	0	0	0	0	0	0	0	0	0	0	0	0	0	0	0
KIYOTAKE-013	B1	1,358,116	205,075,516	29	73	510618	4956346	S	S	S	S	S	S	S	S	S	S	S	0	0	0	0	0	0	0	0	0	0	0	0	0	0	0	0	0	0	0	0	0	0	0	0	0	0	0	0	0	0
KIYOTAKE-014	B1	1,254,320	189,402,320	26	77	396616	4677673	S	S	S	S	S	S	S	S	S	S	S	0	0	0	0	0	0	0	0	0	0	0	0	0	0	0	0	0	0	0	0	0	0	0	0	0	0	0	0	0	0
KIYOTAKE-015	B1	1,277,776	192,944,176	31	54	519467	4667972	S	S	S	S	S	S	S	S	S	S	S	0	0	0	0	0	0	0	0	0	0	0	0	0	0	0	0	0	0	0	0	0	0	0	0	0	0	0	0	0	0
KIYOTAKE-020	B1	1,289,994	194,789,094	35	114	526729	5132425	S	S	S	S	S	S	S	S	S	S	S	0	0	0	0	0	0	0	0	0	0	0	0	0	0	0	0	0	0	0	0	0	0	0	0	0	0	0	0	0	0
KIYOTAKE-021	B1	1,331,710	201,088,210	35	118	656822	5134126	S	S	S	S	S	S	S	S	S	S	S	0	0	0	0	0	0	0	0	0	0	0	0	0	0	0	0	0	0	0	0	0	0	0	0	0	0	0	0	0	0
KIYOTAKE-023	A	1,416,902	213,952,202	34	164	381866	5135202	S	S	S	S	S	S	S	S	S	S	R	0	0	0	0	0	0	0	0	0	0	0	0	1	1	0	1	0	0	0	0	0	0	0	0	0	0	0	0	0	0
KIYOTAKE-024	E	1,211,112	182,877,912	30	96	807512	5202846	S	S	S	S	S	S	S	S	S	S	S	0	0	0	0	0	0	0	0	0	0	0	0	0	0	0	0	0	0	0	0	0	0	0	0	0	0	0	0	0	0
KIYOTAKE-025	B1	1,430,826	216,054,726	35	83	619620	5013698	S	S	S	S	S	S	S	S	S	S	S	0	0	0	0	0	0	0	0	0	0	0	0	0	0	0	0	0	0	0	0	0	0	0	0	0	0	0	0	0	0
KIYOTAKE-026	E	1,265,570	191,101,070	31	87	775869	5135353	S	S	S	S	S	S	S	S	S	S	S	0	0	0	0	0	0	0	0	0	0	0	0	0	0	0	0	0	0	0	0	0	0	0	0	0	0	0	0	0	0
KIYOTAKE-027	B1	1,183,524	178,712,124	31	116	426970	4860545	S	S	S	S	S	S	S	S	S	S	S	0	0	0	0	0	0	0	0	0	0	0	0	0	0	0	0	0	0	0	0	0	0	0	0	0	0	0	0	0	0
KIYOTAKE-030	A	1,227,646	185,374,546	37	97	315115	4833344	S	S	S	S	S	S	S	S	S	S	S	0	0	0	0	0	0	0	0	0	0	0	0	0	0	0	0	0	0	0	0	0	0	0	0	0	0	0	0	0	0
KIYOTAKE-031	E	1,251,600	188,991,600	32	104	879412	5045230	S	S	S	S	S	S	S	S	S	S	S	0	0	0	0	0	0	0	0	0	0	0	0	0	0	0	0	0	0	0	0	0	0	0	0	0	0	0	0	0	0
KIYOTAKE-032	E	1,504,888	227,238,088	39	85	673677	5203045	S	S	S	S	S	S	S	S	S	S	S	0	0	0	0	0	0	0	0	0	0	0	0	0	0	0	0	0	0	0	0	0	0	0	0	0	0	0	0	0	0
KIYOTAKE-033	E	1,181,228	178,365,428	30	86	807695	5202697	S	S	S	S	S	S	S	S	S	S	S	0	0	0	0	0	0	0	0	0	0	0	0	0	0	0	0	0	0	0	0	0	0	0	0	0	0	0	0	0	0
KIYOTAKE-034	B1	1,318,382	199,075,682	36	95	476143	5035044	S	S	S	S	S	S	S	S	S	S	R	0	0	0	0	0	0	1	0	1	0	0	0	0	0	1	0	0	0	0	0	0	0	0	0	0	0	0	1	0	0
KIYOTAKE-035	B1	1,378,606	208,169,506	38	115	568585	5089405	S	S	S	S	S	S	S	S	S	S	R	0	0	0	0	1	0	0	0	1	0	0	0	0	0	1	0	0	0	1	0	1	0	1	0	0	0	0	0	0	1
KIYOTAKE-036	E	1,470,458	222,039,158	41	56	697203	5067022	S	S	S	S	S	S	S	S	S	S	S	0	0	0	0	0	0	0	0	0	0	0	0	0	0	0	0	0	0	0	0	0	0	0	0	0	0	0	0	0	0
KIYOTAKE-037	B1	1,297,522	195,925,822	32	104	396925	4735841	S	S	S	S	S	S	S	S	S	S	S	0	0	0	0	0	0	0	0	0	0	0	0	0	0	0	0	0	0	0	0	0	0	0	0	0	0	0	0	0	0
KIYOTAKE-038	B2	1,386,456	209,354,856	35	183	313436	4912125	S	S	S	S	S	S	S	S	S	S	S	0	0	0	0	0	0	0	0	0	0	0	0	0	0	0	0	0	0	0	0	0	0	0	0	0	0	0	0	0	0
KIYOTAKE-039	D	1,250,114	188,767,214	30	160	565011	5115451	S	S	S	S	S	S	S	S	S	S	S	0	0	0	0	0	0	0	0	0	0	0	0	0	0	0	0	0	0	0	0	0	0	0	0	0	0	0	0	0	0
KIYOTAKE-041	D	1,374,042	207,480,342	37	134	478382	5149024	S	S	S	S	S	S	S	S	S	S	S	0	0	0	0	0	0	0	0	0	0	0	0	0	0	0	0	0	0	0	0	0	0	0	0	0	0	0	0	0	0
KIYOTAKE-043	cladeV	1,339,634	202,284,734	35	106	465084	5158775	S	S	S	S	S	S	S	S	S	S	S	0	0	0	0	0	0	0	0	0	0	0	0	0	0	0	0	0	0	0	0	0	0	0	0	0	0	0	0	0	0
KIYOTAKE-044	E	1,182,050	178,489,550	30	79	777588	5134299	S	S	S	S	S	S	S	S	S	S	S	0	0	0	0	0	0	0	0	0	0	0	0	0	0	0	0	0	0	0	0	0	0	0	0	0	0	0	0	0	0
KIYOTAKE-047	B2	1,234,220	186,367,220	34	59	1237862	5110262	S	S	S	S	S	S	S	S	S	S	S	0	0	0	0	0	0	0	0	0	0	0	0	0	0	0	0	0	0	0	0	0	0	0	0	0	0	0	0	0	0
KIYOTAKE-049	D	1,444,448	218,111,648	42	76	624135	4943828	S	S	S	S	S	S	S	S	S	S	S	0	0	0	0	0	0	0	0	0	0	0	0	0	0	0	0	0	0	0	0	0	0	0	0	0	0	0	0	0	0
KIYOTAKE-050	C	1,428,374	215,684,474	30	126	363625	5095588	S	S	S	S	S	S	S	S	S	S	S	0	0	0	0	0	0	0	0	0	0	0	0	0	0	0	0	0	0	0	0	0	0	0	0	0	0	0	0	0	0
KIYOTAKE-051	cladeV	1,316,300	198,761,300	34	108	663782	5158107	S	S	S	S	S	S	S	S	S	S	S	0	0	0	0	0	0	0	0	0	0	0	0	0	0	0	0	0	0	0	0	0	0	0	0	0	0	0	0	0	0
KIYOTAKE-052	B2	1,241,620	187,484,620	31	136	563002	4878322	S	S	S	S	S	S	S	S	S	S	S	0	0	0	0	0	0	0	0	0	0	0	0	0	0	0	0	0	0	0	0	0	0	0	0	0	0	0	0	0	0
KIYOTAKE-054	cladeV	1,450,868	219,081,068	39	120	872215	5162478	S	S	S	S	S	S	S	S	S	S	S	0	0	0	0	0	0	0	0	0	0	0	0	0	0	0	0	0	0	0	0	0	0	0	0	0	0	0	0	0	0
KIYOTAKE-055	B2	1,457,294	220,051,394	41	150	342122	5068374	S	S	S	S	S	S	S	S	S	S	S	0	0	0	0	0	0	0	0	0	0	0	0	0	0	0	0	0	0	0	0	0	0	0	0	0	0	0	0	0	0
KIYOTAKE-056	B2	1,255,182	189,532,482	38	18	1202513	4692612	S	S	S	S	S	S	S	S	S	S	S	0	0	0	0	0	0	0	0	0	0	0	0	0	0	0	0	0	0	0	0	0	0	0	0	0	0	0	0	0	0
KIYOTAKE-057	B1	1,136,416	171,598,816	33	101	442338	4969346	S	S	S	S	S	S	S	S	S	S	S	0	0	0	0	0	0	0	0	0	0	0	0	0	0	0	0	0	0	0	0	0	0	0	0	0	0	0	0	0	0
KIYOTAKE-058	cladeV	1,543,534	233,073,634	43	113	349180	5159299	S	S	S	S	S	S	S	S	S	S	S	0	0	0	0	0	0	0	0	0	0	0	0	0	0	0	0	0	0	0	0	0	0	0	0	0	0	0	0	0	0
KIYOTAKE-061	B1	1,676,868	253,207,068	52	46	610475	4700643	S	S	S	S	S	S	S	S	S	S	S	0	0	0	0	0	0	0	0	0	0	0	0	0	0	0	0	0	0	0	0	0	0	0	0	0	0	0	0	0	0
KIYOTAKE-062	D	1,335,724	201,694,324	36	125	686465	5399870	S	S	S	S	S	S	S	S	S	S	S	0	0	0	0	0	0	0	0	0	0	0	0	0	0	0	0	0	0	0	0	0	0	0	0	0	0	0	0	0	0

KIYOTAKE-063	B1	1,615,166	243,890,066	48	61	925660	4852843	S	S	S	S	S	S	S	S	S	S	S	0	0	0	0	0	0	0	0	0	0	0	0	0	0	0	0	0	0	0	0	0	0	0	0	0	0	0	0	0	0
KIYOTAKE-065	B1	1,395,866	210,775,766	40	47	705695	4915112	S	S	R	R	S	S	S	S	S	S	S	0	1	0	0	0	0	1	0	0	0	0	0	0	0	0	0	0	0	0	0	0	0	0	0	0	0	0	0	0	0
KIYOTAKE-066	B1	1,277,676	192,929,076	35	123	746231	5183974	S	S	S	S	S	S	S	S	S	S	R	0	0	0	0	0	0	0	0	0	0	0	0	0	0	0	0	0	0	0	0	0	0	0	0	0	0	0	0	0	0
KIYOTAKE-067	F	1,275,590	192,614,090	37	35	757049	4938339	S	S	S	S	S	S	S	S	S	S	S	0	0	0	0	0	0	0	0	0	0	0	0	0	0	0	0	0	0	0	0	0	0	0	0	0	0	0	0	0	0
KIYOTAKE-068	D	1,630,850	246,258,350	46	68	821395	4936600	S	S	S	S	S	S	S	S	S	S	S	0	0	0	0	0	0	0	0	0	0	0	0	0	0	0	0	0	0	0	0	0	0	0	0	0	0	0	0	0	0
KIYOTAKE-069	B2	1,778,290	268,521,790	56	34	1192994	4565146	S	S	S	S	S	S	S	S	S	S	S	0	0	0	0	0	0	0	0	0	0	0	0	0	0	0	0	0	0	0	0	0	0	0	0	0	0	0	0	0	0
KIYOTAKE-070	A	1,402,740	211,813,740	41	138	336341	4909440	S	S	S	S	S	S	S	S	S	S	S	0	0	0	0	0	0	0	0	0	0	0	0	0	0	0	0	0	0	0	0	0	0	0	0	0	0	0	0	0	0
KIYOTAKE-071	B2	1,358,184	205,085,784	39	123	392335	4981468	S	S	S	S	S	S	S	S	S	S	S	0	0	0	0	0	0	0	0	0	0	0	0	0	0	0	0	0	0	0	0	0	0	0	0	0	0	0	0	0	0
KIYOTAKE-072	F	1,406,752	212,419,552	41	36	1244775	4784552	S	S	S	S	S	S	S	S	S	S	S	0	0	0	0	0	0	0	0	0	0	0	0	0	0	0	0	0	0	0	0	0	0	0	0	0	0	0	0	0	0
KIYOTAKE-073	D	1,767,454	266,885,554	52	53	521512	4871780	S	S	S	S	S	S	S	S	S	S	S	0	0	0	0	0	0	0	0	0	0	0	0	0	0	0	0	0	0	0	0	0	0	0	0	0	0	0	0	0	0
KIYOTAKE-074	B1	1,560,008	235,561,208	43	116	408896	5245390	S	S	S	S	S	S	S	S	S	S	S	0	0	0	0	0	0	0	0	0	0	0	0	0	0	0	0	0	0	0	0	0	0	0	0	0	0	0	0	0	0
KIYOTAKE-075	D	1,422,520	214,800,520	42	64	648518	4854264	S	S	S	S	S	S	S	S	S	S	S	0	0	0	0	0	0	0	0	0	0	0	0	0	0	0	0	0	0	0	0	0	0	0	0	0	0	0	0	0	0
KIYOTAKE-076	cladeV	1,524,544	230,206,144	47	70	509532	4614155	S	S	S	S	S	S	S	S	S	S	S	0	0	0	0	0	0	0	0	0	0	0	0	0	0	0	0	0	0	0	0	0	0	0	0	0	0	0	0	0	0
KIYOTAKE-077	cladeV	1,621,594	244,860,694	49	104	1004214	4765818	S	S	S	S	S	S	S	S	S	S	S	0	0	0	0	0	0	0	0	0	0	0	0	0	0	0	0	0	0	0	0	0	0	0	0	0	0	0	0	0	0
KIYOTAKE-078	cladeV	1,332,390	201,190,890	37	112	665384	5158601	S	S	S	S	S	S	S	S	S	S	S	0	0	0	0	0	0	0	0	0	0	0	0	0	0	0	0	0	0	0	0	0	0	0	0	0	0	0	0	0	0
KIYOTAKE-079	A	1,487,940	224,678,940	42	191	272378	5108263	S	S	S	S	S	S	S	S	S	S	S	0	0	0	0	0	0	0	0	0	0	0	0	0	0	0	0	0	0	0	0	0	0	0	0	0	0	0	0	0	0
KIYOTAKE-080	B2	1,659,976	250,656,376	48	91	1189565	4970665	S	S	S	S	S	S	S	S	S	S	S	0	0	0	0	0	0	0	0	0	0	0	0	0	0	0	0	0	0	0	0	0	0	0	0	0	0	0	0	0	0
KIYOTAKE-082	cladeV	1,324,084	199,936,684	37	112	472112	5163139	S	S	S	S	S	S	S	S	S	S	S	0	0	0	0	0	0	0	0	0	0	0	0	0	0	0	0	0	0	0	0	0	0	0	0	0	0	0	0	0	0
KIYOTAKE-085	C	1,398,624	211,192,224	40	49	769127	4924703	S	S	S	S	S	S	S	S	S	S	S	0	0	0	0	0	0	0	0	0	0	0	0	0	0	0	0	0	0	0	0	0	0	0	0	0	0	0	0	0	0
KIYOTAKE-086	cladeV	1,635,054	246,893,154	41	172	304328	5254229	S	S	S	S	S	S	S	S	S	S	S	0	0	0	0	0	0	0	0	0	0	0	0	0	0	0	0	0	0	0	0	0	0	0	0	0	0	0	0	0	0
KIYOTAKE-087	B1	1,394,844	210,621,444	38	46	759643	4968447	S	S	S	S	S	S	S	S	S	S	S	0	0	0	0	0	0	0	0	0	0	0	0	0	0	0	0	0	0	0	0	0	0	0	0	0	0	0	0	0	0
KIYOTAKE-088	B1	1,447,766	218,612,666	42	111	561043	4863885	S	S	S	S	S	S	S	S	S	S	S	0	0	0	0	0	0	0	0	0	0	0	0	0	0	0	0	0	0	0	0	0	0	0	0	0	0	0	0	0	0
KIYOTAKE-089	B2	1,510,672	228,111,472	45	48	681295	4731205	S	S	S	S	S	S	S	S	S	S	S	0	0	0	0	0	0	0	0	0	0	0	0	0	0	0	0	0	0	0	0	0	0	0	0	0	0	0	0	0	0
KIYOTAKE-091	E	1,541,140	232,712,140	42	107	732301	5223917	S	S	S	S	S	S	S	S	S	S	S	0	0	0	0	0	0	0	0	0	0	0	0	0	0	0	0	0	0	0	0	0	0	0	0	0	0	0	0	0	0
KIYOTAKE-093	D	1,524,346	230,176,246	41	76	594554	5273117	S	S	S	S	S	S	S	S	S	S	S	0	0	0	0	0	0	0	0	0	0	0	0	0	0	0	0	0	0	0	0	0	0	0	0	0	0	0	0	0	0
KIYOTAKE-094	D	1,440,156	217,463,556	43	42	637965	4780475	S	S	S	S	S	S	S	S	S	S	S	0	0	0	0	0	0	0	0	0	0	0	0	0	0	0	0	0	0	0	0	0	0	0	0	0	0	0	0	0	0
KIYOTAKE-096	B2	1,534,658	231,733,358	45	55	843817	4854108	S	S	S	S	S	S	S	S	S	S	S	0	0	0	0	0	0	0	0	0	0	0	0	0	0	0	0	0	0	0	0	0	0	0	0	0	0	0	0	0	0
KIYOTAKE-097	A	1,315,478	198,637,178	40	117	351080	4743811	S	S	S	S	S	S	S	S	S	S	S	0	0	0	0	0	0	0	0	0	0	0	0	0	0	0	0	0	0	0	0	0	0	0	0	0	0	0	0	0	0
KIYOTAKE-098	D	1,173,690	177,227,190	30	125	556625	5423903	S	S	S	S	S	S	S	S	S	S	S	0	0	0	0	0	0	0	0	0	0	0	0	0	0	0	0	0	0	0	0	0	0	0	0	0	0	0	0	0	0
KIYOTAKE-099	B2	1,519,846	229,496,746	45	46	653197	4732292	S	S	S	S	S	S	S	S	S	S	S	0	0	0	0	0	0	0	0	0	0	0	0	0	0	0	0	0	0	0	0	0	0	0	0	0	0	0	0	0	0
KIYOTAKE-101	cladeV	1,512,294	228,356,394	46	82	643317	4654583	S	S	S	S	S	S	S	S	S	S	S	0	0	0	0	0	0	0	0	0	0	0	0	0	0	0	0	0	0	0	0	0	0	0	0	0	0	0	0	0	0
KIYOTAKE-102	D	1,426,604	215,417,204	42	46	637982	4872224	S	S	S	S	S	S	S	S	S	S	S	0	0	0	0	0	0	0	0	0	0	0	0	0	0	0	0	0	0	0	0	0	0	0	0	0	0	0	0	0	0
SewageA-001	B2	1,342,844	202,769,444	38	72	613436	4995050	S	S	S	S	S	S	S	S	S	S	S	0	0	0	0	0	0	0	0	0	0	0	0	0	0	0	0	0	0	0	0	0	0	0	0	0	0	0	0	0	0
SewageA-003	B2	1,714,620	258,907,620	44	63	706960	4837508	S	S	S	S	S	S	S	S	S	R	S	0	0	0	0	0	0	0	0	0	0	0	0	0	0	0	0	0	0	0	0	0	0	0	0	0	0	0	0	0	0
SewageA-004	B2	1,485,686	224,338,586	39	89	631455	5195658	S	S	S	S	S	S	S	S	S	S	S	0	0	0	0	0	1	0	0	0	0	0	0	0	0	0	0	0	1	0	0	0	0	0	0	0	0	0	0	0	0
SewageA-005	A	1,515,422	228,828,722	42	135	410951	5059424	S	S	S	S	S	S	S	S	S	S	S	0	0	0	0	0	0	0	0	0	0	0	0	0	0	0	0	0	0	0	0	0	0	0	0	0	0	0	0	0	0
SewageA-006	D	1,305,762	197,170,062	36	99	677132	5214082	S	S	S	S	S	S	S	S	S	S	R	0	0	0	0	0	0	0	0	0	0	0	0	0	0	1	0	0	0	0	0	0	0	0	0	0	0	0	0	0	0
SewageA-007	B2	1,483,258	223,971,958	43	53	914376	4980812	S	S	S	S	S	S	S	S	S	S	S	0	0	0	0	0	0	0	0	0	0	0	0	0	0	0	0	0	0	0	0	0	0	0	0	0	0	0	0	0	0
SewageA-008	A	1,244,546	187,926,446	39	48	763921	4520070	S	S	S	S	S	S	S	S	S	S	S	0	0	0	0	0	0	0	0	0	0	0	0	0	0	0	0	0	0	0	0	0	0	0	0	0	0	0	0	0	0
SewageA-011	D	1,363,466	205,883,366	38	89	336379	5066765	S	S	R	S	R	S	S	S	R	S	S	0	0	0	1	0	0	0	0	1	0	0	0	1	1	0	0	0	0	0	0	0	0	0	0	1	0	1	1	1	0
SewageA-012	B2	1,598,234	241,333,334	42	125	392115	5177188	R	S	S	S	S	S	S	S	S	S	R	0	0	0	0	0	0	1	0	0	0	0	0	0	0	1	0	0	0	0	0	0	0	0	0	0	0	0	0	1	0
SewageA-013	D	1,422,782	214,840,082	41	101	552734	5011765	S	S	S	S	S	S	S	S	S	S	R	0	0	0	0	0	0	1	0	0	0	0	0	1	0	1	0	0	0	0	0	0	0	0	0	0	0	0	0	0	0
SewageA-014	A	1,308,218	197,540,918	39	41	760739	4735556	S	S	S	S	S	S	S	S	S	S	S	0	0	0	0	0	0	0	0	0	0	0	0	0	0	0	0	0	0	0	0	0	0	0	0	0	0	0	0	0	0
SewageA-015	B1	1,365,976	206,262,376	40	78	1084478	4851118	S	S	S	S	S	S	S	S	S	S	S	0	0	0	0	0	0	0	0	1	0	0	0	0	0	1	0	0	0	0	0	1	1	0	0	0	0	0	1	1	0
SewageA-016	A	1,566,356	236,519,756	43	74	660019	5269868	S	S	S	S	S	S	S	S	S	S	R	0	0	0	0	0	0	0	0	0	0	0	0	0	0	0	0	0	0	0	0	0	0	0	0	0	0	0	0	0	0
SewageA-018	A	1,572,014	237,374,114	47	110	476922	4751314	S	S	S	S	S	S	S	S	S	S	S	0	0	0	0	0	0	0	0	0	0	0	0	0	0	0	0	0	0	0	0	0	0	0	1	0	0	1	0	0	0
SewageA-019	D	1,366,678	206,368,378	37	165	375775	5318788	S	S	S	S	R	S	S	S	R	R	S	0	1	0	0	0	0	1	0	0	0	0	0	0	0	0	0	0	0	0	0	0	0	0	0	0	0	0	0	0	0
SewageA-020	A	1,436,688	216,939,888	42	117	402478	4790949	S	S	S	S	S	S	S	S	S	S	S	0	0	0	0	0	0	0	0	0	0	0	0	0	0	0	0	0	0	0	0	0	0	0	1	0	0	1	0	0	0
SewageA-021	B2	1,633,478	246,655,178	45	66	539589	5219142	S	S	R	S	R	S	S	S	S	S	S	0	0	0	0	0	0	0	0	0	0	0	0	0	0	0	0	0	0	0	0	0	0	0	0	0	0	0	0	0	0
SewageA-022	B2	1,230,620	185,823,620	36	84	521330	4862916	S	S	S	S	S	S	S	S	S	S	S	0	0	0	0	0	0	1	0	0	0	0	0	0	0	0	0	0	0	0	0	0	0	0	0	0	0	0	0	1	0
SewageA-023	B1	1,159,628	175,103,828	32	69	715504	4771690	S	S	S	S	S	S	S	S	S	S	S	0	0	0	0	0	0	0	0	0	0	0	0	0	0	0	0	0	0	0	0	0	0	0	0	0	0	0	0	0	0
SewageA-024	D	1,638,018	247,340,718	43	114	468232	5281045	S	S	S	S	S	S	S	S	S	R	R	0	0	0	0	0	0	0	0	0	0	0	0	1	1	1	0	0	0	0	0	0	0	0	0	0	0	1	0	0	0
SewageA-025	B1	1,455,386	219,763,286	42	154	905228	4843579	S	S	S	S	S	S	S	S	S	S	S	0	0	0	0	0	0	0	0	0	0	0	0	0	0	0	0	0	0	0	0	0	0	0	0	0	0	0	0	0	0
SewageA-026	B2	1,392,536	210,272,936	38	93	624822	5182915	S	S	S	S	S	S	S	S	S	S	R	0	0	0	0	0	0	0	0	0	0	0	0	0	0	0	0	0	0	0	0	0	0	0	0	0	0	0	0	0	0
SewageA-027	B1	1,462,250	220,799,750	43	73	516084	4810920	S	S	S	S	S	S	S	S	S	S	S	0	0	0	0	0	0	0	0	0	0	0	0	0	0	0	0	0	0	0	0	0	0	0	0	0	0	0	0	0	0
SewageA-028	A	1,305,334	197,105,434	39	108	531544	4759087	S	S	S	S	S	S	S	S	S	S	S	0	0	0	0	0	0	0	0	0	0	0	0	0	0	0	0	0	0	0	0	0	0	0	0	0	0	1	0	0	0
SewageA-029	B2	1,579,480	238,501,480	47	60	742523	4846040	S	S	S	S	S	S	S	S	S	S	S	0	0	0	0	0	0	0	0	0	0	0	0	0	0	0	0	0	0	0	0	0	0	0	0	0	0	0	0	0	0
SewageA-030	D	1,476,510	222,953,010	45	55	512219	4711937	S	S	S	S	S	S	S	S	S	S	S	0	0	0	0	0	0	0	0	0	0	0	0	0	0	0	0	0	0	0	0	0	0	0	0	0	0	0	0	0	0
SewageA-032	B1	1,505,488	227,328,688	46	53	885657	4662949	S	S	S	S	S	S	S	S	S	S	S	0	0	0	0	0	0	0	0	0	0	0	0	0	0	0	0	0	0	0	0	0	0	0	0	0	0	0	0	0	0
SewageA-033	B2	1,376,434	207,841,534	39	62	521206	4840103	S	S	S	S	S	S	S	S	S	S	S	0	0	0	0	0	0	0	0	0	0	0	0	0	0	0	0	0	0	0	0	0	0	0	0	0	0	0	0	0	0
SewageA-034	B2	1,438,604	217,229,204	39	84	539585	5217916	S	S	S	S	S	S	S	S	S	S	S	0	0	0	0	0	0	0	0	0	0	0	0	0	0	0	0	0	0	0	0	0	0	0	0	0	0	0	0	0	0
SewageA-036	B1	1,380,982	208,528,282	43	64	1139462	4614795	S	S	S	S	S	S	S	S	S	R	R	0	0	0	0	0	0	0	0	0	0	0	0	0	0	0	0	0	0	0	0	0	0	0	0	0	0	0	0	0	0
SewageA-038	B1	1,386,952	209,429,752	41	75	427208	4866883	S	S	S	S	S	S	S	S	S	S	R	0	0	0	0	0	0	0	0	0	0	0	0	0	0	0	0	0	0	0	0	0	0	0	0	0	0	0	0	0	0
SewageA-039	A	1,334,518	201,512,218	39	75	590274	4734595	S	S	S	S	S	S	S	S	S	S	R	0	0	0	0	0	0	1	0	0	1	0	0	1	1	1	0	0	0	0	0	0	0	0	1	0	0	1	1	1	0
SewageA-040	B1	1,375,754	207,738,854	40	66	642961	4973261	R	S	S	S	S	S	S	S	R	S	S	0	0	0	0	0	0	1	0	0	0	0	0	1	1	1	0	0	0	0	0	0	0	1	0	0	0	1	0	0	0
SewageA-041	D	1,111,084	167,773,684	33	30	781915	4801816	S	S	S	S	S	S	S	S	S	S	S	0	0	0	0	0	0	0	0	0	0	0	0	0	0	0	0	0	0	0	0	0	0	0	0	0	0	0	0	0	0
SewageA-042	B2	1,700,792	256,819,592	44	59	706961	4838466	S	S	S	S	S	S	S	S	S	S	S	0	0	0	0	0	0	0	0	0	0	0	0	0	0	0	0	0	0	0	0	0	0	0	0	0	0	0	0	0	0
SewageA-043	B2	1,226,354	185,179,454	34	57	706964	4839625	S	S	S	S	S	S	S	S	S	S	S	0	0	0	0	0	0	0	0	0	0	0	0	0	0	0	0	0	0	0	0	0	0	0	0	0	0	0	0	0	0
SewageA-044	B2	1,463,978	221,060,678	41	33	1005375	4913156	S	S	S	S	S	S	S	S	S	S	S	0	0	0	0	0	0	0	0	0	0	0	0	0	0	0	0	0	0	0	0	0	0	0	0	0	0	0	0	0	0
SewageA-046	A	1,261,126	190,430,026	36	123	411060	5063237	S	S	S	S	S	S	S	S	S	S	S	0	0	0	0	0	0	0	0	0	0	0	0	0	0	0	0	0	0	0	0	0	0	0	0	0	0	0	0	0	0
SewageA-047	A	1,101,600	166,341,600	34	76	569789	4727164	S	S	S	S	S	S	S	S	S	S	S	0	0	0	0	0	0	0	0	0	0	0	0	0	0	0	0	0	0	0	0	0	0	0	0	0	0	0	0	0	0
SewageA-049	B2	1,360,416	205,422,816	33	63	827735	5038733	S	S	S	S	S	S	S	S	S	S	S	0	0	0	0	0	0	0	0	0	0	0	0	0	0	0	0	0	0	0	0	0	0	0	0	0	0	0	0	0	0
SewageA-051	B1	1,415,248	213,702,448	36	150	429960	5041035	S	S	S	S	S	S	S	S	S	S	S	0	0	0	0	0	0	0	0	0	0	0	0	0	0	0	0	0	0	0	0	0	0	0	0	0	0	0	0	0	0
SewageA-052	B2	1,174,288	177,317,488	32	62	521310	4842300	S	S	S	S	S	S	S	S	S	S	S	0	0	0	0	0	0	0	0	0	0	0	0	0	0	0	0	0	0	0	0	0	0	0	0	0	0	0	0	0	0
SewageA-054	B1	1,427,834	215,602,934	39	70	513488	4775043	S	S	S	S	S	S	S	S	S	S	S	0	0	0	0	0	0	0	0	0	0	0	0	0	0	0	0	0	0	0	0	0	0	0	0	0	0	0	0	0	0
SewageA-055	B2	1,259,910	190,246,410	37	61	706888	4839604	S	S	S	S	S	S	S	S	S	S	S	0	0	0	0	0	0	0	0	0	0	0	0	0	0	0	0	0	0	0	0	0	0	0	0	0	0	0	0	0	0
SewageA-056	B2	1,258,714	190,065,814	31	322	588319	4873454	S	S	S	S	S	S	S	S	S	S	S	0	0	0	0	0	0	0	0	0	0	0	0	0	0	0	0	0	0	0	1	0	0	0	0	0	0	0	0	0	0
SewageA-057	A	1,150,280	173,692,280	31	217	271106	4974190	S	S	S	S	S	S	S	S	S	S	S	0	0	0	0	0	0	0	0	0	0	0	0	0	0	0	0	0	0	0	0	0	0	0	0	0	0	0	0	0	0
SewageA-058	B2	1,391,952	210,184,752	40	36	738632	4913079	S	S	S	S	S	S	S	S	S	S	S	0	0	0	0	0	0	0	0	0	0	0	0	0	0	0	0	0	0	0	0	0	0	0	0	0	0	0	0	0	0
SewageA-059	B1	1,533,598	231,573,298	46	58	721001	4736998	S	S	S	S	S	S	S	S	S	S	S	0	0	0	0	0	0	0	0	0	0	0	0	0	0	0	0	0	0	0	0	0	0	0	0	0	0	0	0	0	0
SewageA-064	A	1,243,922	187,832,222	36	98	569868	4662513	S	S	S	S	S	S	S	S	S	S	S	0	0	0	0	0	0	0	0	0	0	0	0	0	0	0	0	0	0	0	0	0	0	0	0	0	0	0	0	0	0
SewageA-065	B2	1,406,744	212,418,344	40	122	517431	4994490	R	S	S	S	S	S	S	S	S	S	R	0	0	0	0	0	0	1	0	0	1	0	0	1	1	1	0	0	0	0	0	0	0	0	0	0	0	1	1	1	0
SewageA-066	F	1,400,298	211,444,998	40	59	716332	5023414	S	S	R	S	R	S	S	S	S	S	S	0	0	1	0	0	0	0	0	0	0	0	0	0	0	0	0	0	0	0	0	0	0	0	0	0	0	0	0	0	0
SewageA-068	B2	1,251,824	189,025,424	35	53	902431	5139537	S	S	S	S	S	S	S	S	S	S	S	0	0	0	0	0	0	0	0	0	0	0	0	0	0	0	0	0	0	0	0	0	0	0	0	0	0	0	0	0	0
SewageA-069	B2	1,344,420	203,007,420	37	96	620054	5270894	S	S	S	S	S	S	S	S	S	S	S	0	0	0	0	0	0	0	0	0	0	0	0	0	0	0	0	0	0	0	0	0	0	0	0	0	0	0	0	0	0
SewageA-073	B2	1,262,592	190,651,392	35	64	1214588	4998502	S	S	S	S	S	S	S	S	S	S	S	0	0	0	0	0	0	0	0	0	0	0	0	0	0	0	0	0	0	0	0	0	0	0	0	0	0	0	0	0	0
SewageA-074	B2	1,590,332	240,140,132	42	99	619997	5157828	S	S	S	S	S	S	S	S	S	S	S	0	0	0	0	0	0	0	0	0	0	0	0	0	0	0	0	0	0	0	0	0	0	0	0	0	0	0	0	0	0
SewageA-076	B1	1,339,932	202,329,732	31	81	702732	4802684	S	S	S	S	S	S	S	S	S	S	S	0	0	0	0	0	0	0	0	0	0	0	0	0	0	0	0	0	0	0	0	0	0	0	0	0	0	0	0	0	0
SewageA-077	B1	1,298,026	196,001,926	29	78	514299	4711536	S	S	S	S	S	S	S	S	S	S	S	0	0	0	0	0	0	0	0	0	0	0	0	0	0	0	0	0	0	0	0	0	0	0	0	0	0	0	0	0	0
SewageA-079	C	1,207,504	182,333,104	30	99	496992	5117476	S	S	S	S	S	S	S	S	S	S	S	0	0	0	0	0	0	0	0	0	0	0	0	0	0	0	0	0	0	0	0	0	0	0	0	0	0	0	0	0	0
SewageA-080	B1	1,248,908	188,585,108	38	55	622933	4634223	S	S	S	S	S	S	S	S	S	S	S	0	0	0	0	0	0	0	0	0	0	0	0	0	0	0	0	0	0	0	0	0	0	0	0	0	0	0	0	0	0
SewageA-081	B1	1,436,506	216,912,406	41	55	629052	4773980	S	S	S	S	S	S	S	S	S	S	S	0	0	0	0	0	0	0	0	0	0	0	0	0	0	0	0	0	0	0	0	0	0	0	0	0	0	0	0	0	0
SewageA-082	B2	1,349,784	203,817,384	36	75	707418	4877065	S	S	S	S	S	S	S	S	S	S	S	0	0	0	0	0	0	0	0	0	0	0	0	0	0	0	0	0	0	0	0	0	0	0	0	0	0	0	0	0	0
SewageA-083	B2	1,236,418	186,699,118	34	71	626953	5172626	S	S	S	S	S	S	S	S	S	S	S	0	0	0	0	0	0	0	0	0	0	0	0	0	0	0	0	0	0	0	0	0	0	0	0	0	0	0	0	0	0
SewageA-084	B1	858,880	129,690,880	24	88	568575	4860431	S	S	S	S	S	S	S	S	S	S	S	0	0	0	0	0	0	0	0	0	0	0	0	0	0	0	0	0	0	0	0	0	0	0	0	0	0	0	0	0	0
SewageA-085	E	1,472,012	222,273,812	42	56	996289	5069318	S	S	S	S	S	S	S	S	S	S	S	0	0	0	0	0	0	0	0	0	0	0	0	0	0	0	0	0	0	0	0	0	0	0	0	0	0	0	0	0	0
SewageA-086	B1	1,554,856	234,783,256	48	64	568448	4697099	S	S	S	S	S	S	S	S	S	S	S	0	0	0	0	0	0	0	0	0	0	0	0	0	0	0	0	0	0	0	0	0	0	0	0	0	0	0	0	0	0
SewageA-088	B2	1,849,250	279,236,750	43	74	379697	4904481	S	S	S	S	S	S	S	S	S	S	S	0	0	0	0	0	0	0	0	0	0	0	0	0	0	0	0	0	0	0	0	0	0	0	0	0	0	0	0	0	0
SewageA-089	A	1,164,128	175,783,328	31	137	309120	5056057	S	S	S	S	S	S	S	S	S	S	S	0	0	0	0	0	0	0	0	0	0	0	0	0	0	0	0	0	0	0	0	0	0	0	0	0	0	0	0	0	0
SewageA-092	A	1,487,136	224,557,536	43	133	251804	4948029	S	S	S	S	S	S	S	S	S	S	S	0	0	0	0	0	0	0	0	0	0	0	0	0	0	0	0	0	0	0	0	0	0	0	0	0	0	0	0	0	0
SewageA-095	A	1,271,412	191,983,212	32	104	339682	4499909	S	S	S	S	S	S	S	S	S	S	S	0	0	0	0	0	0	0	0	0	0	0	0	0	0	0	0	0	0	0	0	0	0	0	0	0	0	0	0	0	0
SewageA-097	D	1,278,334	193,028,434	28	64	690845	4955064	S	S	S	S	S	S	S	S	S	S	R	0	0	0	0	0	0	0	0	0	0	0	0	0	0	0	0	1	0	0	0	0	0	0	0	0	0	0	0	0	0
SewageA-098	B2	1,110,312	167,657,112	27	65	521368	4838616	S	S	S	S	S	S	S	S	S	S	S	0	0	0	0	0	0	0	0	0	0	0	0	0	0	0	0	0	0	0	0	0	0	0	0	0	0	0	0	0	0
SewageA-100	D	1,019,992	154,018,792	28	61	895389	4968867	S	S	S	S	S	S	S	S	S	S	S	0	0	0	0	0	0	0	0	0	0	0	0	0	0	0	0	0	0	0	0	0	0	0	0	0	0	0	0	0	0

SewageB-001	B2	1,211,624	182,955,224	31	102	860658	4946377	R	S	S	S	S	S	S	S	S	S	S	0	0	0	0	0	0	1	0	0	0	0	0	0	0	0	0	0	0	0	0	0	0	0	0	0	0	0	0	1	0
SewageB-002	B2	1,470,254	222,008,354	41	34	782981	4687100	S	S	S	S	S	S	S	S	S	S	S	0	0	0	0	0	0	0	0	0	0	0	0	0	0	0	0	0	0	0	0	0	0	0	0	0	0	0	0	0	0
SewageB-003	B2	1,261,998	190,561,698	33	105	391392	5062846	R	S	R	S	R	S	S	S	R	S	R	0	0	0	1	0	0	0	0	0	0	0	0	0	0	0	0	0	0	0	0	0	0	0	0	0	0	0	0	0	0
SewageB-004	B2	1,315,542	198,646,842	37	22	1964606	4894532	S	S	R	S	S	S	S	S	S	S	S	0	0	0	0	0	0	0	0	0	0	0	0	0	0	0	0	0	0	0	0	0	0	0	0	0	0	0	0	0	0
SewageB-005	A	1,127,846	170,304,746	32	90	545638	4906792	S	S	S	S	S	S	S	S	S	S	S	0	0	0	0	0	0	0	0	0	0	0	0	0	0	0	0	0	0	0	0	0	0	0	0	0	0	0	0	0	0
SewageB-007	B1	1,523,812	230,095,612	36	70	663486	4784334	S	S	S	S	S	S	S	S	S	S	S	0	0	0	0	0	0	0	0	0	0	0	0	0	0	0	0	0	0	0	0	0	0	0	0	0	0	0	0	0	0
SewageB-008	B1	1,383,988	208,982,188	35	129	490664	5135371	S	S	S	S	S	S	S	S	S	S	S	0	0	0	0	0	0	0	0	0	0	0	0	0	0	0	0	0	0	0	0	0	0	0	0	0	0	0	0	0	0
SewageB-009	B1	1,628,452	245,896,252	45	59	603096	4690747	S	S	S	S	S	S	S	S	S	S	S	0	0	0	0	0	0	0	0	0	0	0	0	0	0	0	0	0	0	0	0	0	0	0	0	0	0	0	0	0	0
SewageB-010	B1	1,661,364	250,865,964	47	57	895524	4730016	S	S	S	S	S	S	S	S	S	S	S	0	0	0	0	0	0	0	0	0	0	0	0	0	0	0	0	0	0	0	0	0	0	0	0	0	0	0	0	0	0
SewageB-011	B1	1,193,166	180,168,066	34	71	629556	4836406	R	S	S	R	R	S	S	R	S	S	R	1	0	0	0	0	0	0	1	1	0	0	0	0	0	1	0	0	0	0	0	0	0	0	0	0	0	0	1	0	0
SewageB-012	B2	744,552	112,427,352	20	93	515770	5220860	S	S	S	S	S	S	S	S	S	S	S	0	0	0	0	0	0	0	0	0	0	0	0	0	0	0	0	0	0	0	0	0	0	0	0	0	0	0	0	0	0
SewageB-013	B2	1,394,532	210,574,332	38	108	665463	5168749	S	S	S	S	S	S	S	S	S	S	S	0	0	0	0	0	0	0	0	0	0	0	0	0	0	0	0	0	0	0	0	0	0	0	0	0	0	0	0	0	0
SewageB-014	B2	1,463,126	220,932,026	38	40	818369	5028867	S	S	S	S	S	S	S	S	S	S	S	0	0	0	0	0	0	0	0	0	0	0	0	0	0	0	0	0	0	0	0	0	0	0	0	0	0	0	0	0	0
SewageB-015	B2	1,261,184	190,438,784	35	51	864325	5119205	S	S	S	S	S	S	S	S	S	S	S	0	0	0	0	0	0	0	0	0	0	0	0	0	0	0	0	0	0	0	0	0	0	0	0	0	0	0	0	0	0
SewageB-016	B2	924,074	139,535,174	26	32	814945	4948845	S	S	S	S	S	S	S	S	S	S	S	0	0	0	0	0	0	0	0	0	0	0	0	0	0	0	0	0	0	0	0	0	0	0	0	0	0	0	0	0	0
SewageB-017	B2	1,149,380	173,556,380	32	34	904550	5013785	S	S	S	S	S	S	S	S	S	S	R	0	0	0	0	0	0	0	0	0	0	0	0	0	0	0	0	0	0	0	0	0	0	0	0	0	0	0	0	0	0
SewageB-018	A	974,530	147,154,030	28	101	309077	4725004	S	S	R	S	S	S	S	S	S	S	R	0	0	0	0	0	0	0	0	0	0	0	0	0	0	1	0	0	0	0	0	0	0	0	0	0	0	0	0	0	0
SewageB-019	D	1,266,630	191,261,130	30	120	467975	5224749	S	S	S	S	S	S	S	S	S	S	S	0	0	0	0	0	0	0	0	0	0	0	0	0	0	0	0	0	0	0	0	0	0	0	0	0	0	0	0	0	0
SewageB-020	B2	1,365,286	206,158,186	35	57	683811	4882878	S	S	S	S	S	S	S	S	S	S	S	0	0	0	0	0	0	0	0	0	0	0	0	0	0	0	0	0	0	0	0	0	0	0	0	0	0	0	0	0	0
SewageB-021	A	1,115,980	168,512,980	31	92	515015	4779611	S	S	S	S	S	S	S	S	S	S	S	0	0	0	0	0	0	0	0	0	0	0	0	0	0	0	0	0	0	0	0	0	0	0	0	0	0	0	0	0	0
SewageB-022	B2	1,150,638	173,746,338	29	38	600990	4814672	S	S	S	S	S	S	S	S	S	S	R	0	0	0	0	0	0	0	0	0	0	0	0	0	0	0	0	0	0	0	0	0	0	0	0	0	0	0	0	0	0
SewageB-023	B2	1,352,704	204,258,304	38	73	537743	5118993	S	S	S	S	S	S	S	S	S	S	R	0	0	0	0	0	0	0	0	0	0	0	0	0	0	0	1	0	0	0	0	0	0	0	0	0	0	0	0	0	0
SewageB-025	A	1,239,832	187,214,632	33	142	296865	4912740	S	S	S	S	S	S	S	S	S	S	S	0	0	0	0	0	0	0	0	0	0	0	0	0	0	0	0	0	0	0	0	0	0	0	0	0	0	0	0	0	0
SewageB-026	B2	1,057,318	159,655,018	29	40	684326	4799134	S	S	S	S	S	S	S	S	S	S	S	0	0	0	0	0	0	0	0	0	0	0	0	0	0	0	0	0	0	0	0	0	0	0	0	0	0	0	0	0	0
SewageB-027	B2	1,584,576	239,270,976	42	67	761134	5153729	S	S	S	S	S	S	S	S	S	S	R	0	0	0	0	0	0	0	0	0	0	0	0	0	0	0	1	0	0	0	0	0	0	0	0	0	0	0	0	0	0
SewageB-028	B1	832,528	125,711,728	24	44	752888	4776218	S	S	S	S	S	S	S	S	S	S	S	0	0	0	0	0	0	0	0	0	0	0	0	0	0	0	0	0	0	0	0	0	0	0	0	0	0	0	0	0	0
SewageB-029	B1	1,001,992	151,300,792	28	57	433093	4684421	S	S	S	S	S	S	S	S	S	S	S	0	0	0	0	0	0	0	0	0	0	0	0	0	0	0	0	0	0	0	0	0	0	0	0	0	0	0	0	0	0
SewageB-030	D	1,105,848	166,983,048	30	135	497070	5199743	S	S	S	S	S	S	S	S	S	S	R	0	0	0	0	0	0	0	0	0	0	0	0	0	0	0	0	0	0	0	0	0	0	0	0	0	0	0	0	0	0
SewageB-031	B1	1,278,044	192,984,644	28	54	427299	4681004	S	S	S	S	S	S	S	S	S	S	R	0	0	0	0	0	0	0	0	0	0	0	0	0	0	0	1	0	0	0	0	0	0	0	0	0	0	0	0	0	0
SewageB-032	A	1,234,918	186,472,618	30	152	296780	4911634	S	S	S	S	S	S	S	S	S	S	S	0	0	0	0	0	0	0	0	0	0	0	0	0	0	0	0	0	0	0	0	0	0	0	0	0	0	0	0	0	0
SewageB-033	A	1,039,028	156,893,228	27	91	545647	4905653	S	S	S	S	S	S	S	S	S	S	S	0	0	0	0	0	0	0	0	0	0	0	0	0	0	0	0	0	0	0	0	0	0	0	0	0	0	0	0	0	0
SewageB-034	D	1,291,658	195,040,358	34	53	910663	5317532	S	S	S	S	S	S	S	S	S	S	S	0	0	0	0	0	0	0	0	0	0	0	0	0	0	0	0	0	0	0	0	0	0	0	0	0	0	0	0	0	0
SewageB-035	B2	1,157,324	174,755,924	32	59	1154414	5125142	S	S	S	S	S	S	S	S	S	S	S	0	0	0	0	0	0	0	0	0	0	0	0	0	0	0	0	0	0	0	0	0	0	0	0	0	0	0	0	0	0
SewageB-037	B2	1,166,934	176,207,034	27	73	532626	5032947	S	S	S	S	S	S	S	S	S	S	S	0	0	0	0	0	0	0	0	0	0	0	0	0	0	0	0	0	0	0	0	0	0	0	0	0	0	0	0	0	0
SewageB-038	A	1,149,740	173,610,740	33	96	306648	4774447	S	S	S	S	S	S	S	S	S	S	R	0	0	0	0	0	0	0	0	0	0	0	0	0	0	1	0	0	0	0	0	0	0	0	0	0	0	0	0	0	0
SewageB-039	B2	1,277,640	192,923,640	35	64	767625	5128489	S	S	S	S	S	S	S	S	R	S	R	0	0	0	0	0	0	0	0	0	0	0	0	0	0	1	0	0	0	0	0	0	0	0	0	0	0	0	0	0	0
SewageB-040	B2	1,161,828	175,436,028	33	39	683842	4829524	S	S	S	S	S	S	S	S	S	S	S	0	0	0	0	0	0	0	0	0	0	0	0	0	0	0	0	0	0	0	0	0	0	0	0	0	0	0	0	0	0
SewageB-041	D	1,454,866	219,684,766	41	68	664390	5122228	S	S	S	S	S	S	S	S	S	S	S	0	0	0	0	0	0	0	0	0	0	0	0	0	0	0	0	0	0	0	0	0	0	0	0	0	0	0	0	0	0
SewageB-042	B2	1,167,052	176,224,852	36	31	1194360	4635516	S	S	S	S	S	S	S	S	S	S	S	0	0	0	0	0	0	0	0	0	0	0	0	0	0	0	0	0	0	0	0	0	0	0	0	0	0	0	0	0	0
SewageB-044	E	1,250,974	188,897,074	30	140	402336	5385985	S	S	S	S	S	S	S	S	R	S	S	0	0	0	0	0	0	0	0	1	0	0	1	0	0	0	0	0	0	0	0	0	0	0	0	0	0	1	0	0	0
SewageB-045	A	1,380,600	208,470,600	37	142	281822	4696389	S	S	S	S	S	S	S	S	S	S	S	0	0	0	0	0	0	0	0	0	0	0	0	1	1	0	0	0	0	0	0	0	0	0	0	0	0	0	0	0	0
SewageB-046	A	1,456,476	219,927,876	40	121	250111	4709857	R	R	S	S	S	S	S	S	S	S	R	0	0	0	0	0	0	1	0	0	0	0	0	0	0	1	0	0	0	0	0	1	0	0	0	0	0	0	0	0	0
SewageB-047	B1	1,263,334	190,763,434	35	71	662457	4891548	S	S	S	S	S	S	S	S	S	S	S	0	0	0	0	0	0	0	0	0	0	0	0	0	0	0	0	0	0	0	0	0	0	0	0	0	0	0	0	0	0
SewageB-048	B2	1,332,682	201,234,982	39	29	1193594	4634109	S	S	S	S	S	S	S	S	S	S	S	0	0	0	0	0	0	0	0	0	0	0	0	0	0	0	0	0	0	0	0	0	0	0	0	0	0	0	0	0	0
SewageB-049	B2	1,132,564	171,017,164	26	93	664316	5198530	S	S	S	S	S	S	S	S	S	S	S	0	0	0	0	0	0	0	0	0	0	0	0	0	0	0	0	0	0	0	0	0	0	0	0	0	0	0	0	0	0
SewageB-050	A	1,243,162	187,717,462	30	113	306712	4772406	S	S	S	S	S	S	S	S	S	S	R	0	0	0	0	0	0	0	0	0	0	0	0	0	0	1	0	0	0	0	0	0	0	0	0	0	0	0	0	0	0
SewageB-051	B2	1,237,780	186,904,780	32	33	641376	4806401	S	S	S	S	S	S	S	S	S	S	S	0	0	0	0	0	0	0	0	0	0	0	0	0	0	0	0	0	0	0	0	0	0	0	0	0	0	0	0	0	0
SewageB-052	A	1,197,880	180,879,880	33	97	306646	4774138	S	S	S	S	S	S	S	S	S	S	R	0	0	0	0	0	0	0	0	0	0	0	0	0	0	1	0	0	0	0	0	0	0	0	0	0	0	0	0	0	0
SewageB-053	B1	1,377,546	208,009,446	39	64	665887	4891925	S	S	S	S	S	S	S	S	S	S	S	0	0	0	0	0	0	0	0	0	0	0	0	0	0	0	0	0	0	0	0	0	0	0	0	0	0	0	0	0	0
SewageB-054	B1	1,485,958	224,379,658	40	61	610008	4674662	S	S	S	S	S	S	S	S	S	S	S	0	0	0	0	0	0	0	0	0	0	0	0	0	0	0	0	0	0	0	0	0	0	0	0	0	0	0	0	0	0
SewageB-055	B1	906,102	136,821,402	25	83	396621	4728229	S	S	S	S	S	S	S	S	S	S	S	0	0	0	0	0	0	0	0	0	0	0	0	0	0	0	0	0	0	0	0	0	0	0	0	0	0	0	0	0	0
SewageB-056	D	1,192,462	180,061,762	32	62	624222	5057035	S	S	S	S	S	S	S	S	S	S	S	0	0	0	0	0	0	0	0	0	0	0	0	0	0	0	0	0	0	0	0	0	0	0	0	0	0	0	0	0	0
SewageB-057	D	1,494,928	225,734,128	43	42	826988	4850361	S	S	S	S	S	S	S	S	S	S	S	0	0	0	0	0	0	0	0	0	0	0	0	0	0	0	0	0	0	0	0	0	0	0	0	0	0	0	0	0	0
SewageB-058	B2	1,543,044	232,999,644	41	110	488927	5076393	S	S	S	S	S	S	S	R	S	S	S	0	0	0	0	0	0	1	0	0	0	0	0	0	0	0	0	0	0	0	0	0	0	0	0	0	0	0	0	0	0
SewageB-059	B2	1,447,032	218,501,832	42	43	684248	4834612	S	S	S	S	S	S	S	S	S	S	S	0	0	0	0	0	0	0	0	0	0	0	0	0	0	0	0	0	0	0	0	0	0	0	0	0	0	0	0	0	0
SewageB-060	B1	1,341,240	202,527,240	40	72	677723	4784025	S	S	S	S	S	S	S	S	S	S	S	0	0	0	0	0	0	0	0	0	0	0	0	0	0	0	0	0	0	0	0	0	0	0	0	0	0	0	0	0	0
SewageB-061	B1	1,171,154	176,844,254	28	112	491157	5039547	S	S	S	S	S	S	S	S	S	S	S	0	0	0	0	0	0	0	0	0	0	0	0	0	0	0	0	0	0	0	0	0	0	0	0	0	0	0	0	0	0
SewageB-063	A	1,570,312	237,117,112	47	151	268266	4584703	S	S	S	S	S	S	S	S	S	S	R	0	0	0	0	0	0	0	0	0	1	0	0	0	0	1	0	0	0	0	0	0	0	0	1	0	0	0	0	0	0
SewageB-064	B1	1,213,752	183,276,552	36	92	373163	4790639	S	S	S	S	S	S	S	S	S	S	S	0	0	0	0	0	0	0	0	0	0	0	0	0	0	0	0	0	0	0	0	0	0	0	0	0	0	0	0	0	0
SewageB-065	A	1,352,462	204,221,762	37	110	410970	5110047	S	S	S	S	S	S	S	S	S	S	S	0	0	0	0	0	0	0	0	0	0	0	0	0	0	0	0	0	0	0	0	0	0	0	0	0	0	0	0	0	0
SewageB-066	E	1,431,222	216,114,522	42	50	1272300	4884025	S	S	S	S	S	S	S	S	S	S	S	0	0	0	0	0	0	0	0	0	0	0	0	0	0	0	0	0	0	0	0	0	0	0	0	0	0	0	0	0	0
SewageB-068	B1	1,322,028	199,626,228	38	88	453885	4830835	R	S	R	S	R	S	S	R	S	S	R	1	0	0	0	0	0	0	1	1	0	0	0	0	0	1	0	0	0	0	0	0	0	0	0	0	0	0	1	0	0
SewageB-070	B2	1,489,614	224,931,714	38	224	707665	5523889	S	S	S	S	S	S	S	S	S	S	S	0	0	0	0	0	0	0	0	0	0	0	0	1	1	0	0	0	0	0	0	0	0	0	0	0	0	0	0	0	0
SewageB-071	B1	1,288,462	194,557,762	35	95	473938	5279501	R	S	S	S	S	S	S	S	S	S	R	0	0	0	0	0	0	1	0	0	1	1	0	1	1	1	1	0	0	0	0	0	0	0	1	0	0	1	1	0	0
SewageB-076	D	1,435,362	216,739,662	40	67	613993	4909982	S	S	S	S	S	S	S	S	S	S	S	0	0	0	0	0	0	0	0	0	0	0	0	0	0	0	0	0	0	0	0	0	0	0	0	0	0	0	0	0	0
SewageB-077	B1	1,275,482	192,597,782	38	67	416883	4826800	S	S	R	S	R	S	S	S	S	S	S	0	0	0	0	0	0	0	0	0	0	0	0	0	0	0	0	0	0	0	0	0	0	0	0	0	0	0	0	0	0
SewageB-080	D	1,368,774	206,684,874	38	83	725388	5188672	S	S	S	S	S	S	S	S	S	S	S	0	0	0	0	0	0	0	0	0	0	0	0	0	0	0	0	0	0	0	0	0	0	0	0	0	0	0	0	0	0
SewageB-082	B2	1,317,534	198,947,634	36	67	1018936	5201192	S	S	S	S	S	S	S	S	S	S	S	0	0	0	0	0	0	0	0	0	0	0	0	0	0	0	0	0	0	0	0	0	0	0	0	0	0	0	0	0	0
SewageB-083	B2	2,575,274	388,866,374	70	94	1037326	5230517	S	S	S	S	S	S	S	S	S	S	S	0	0	0	0	0	0	0	0	0	0	0	0	0	0	0	0	0	0	0	0	0	0	0	0	0	0	0	0	0	0
SewageB-084	B1	1,273,532	192,303,332	38	75	632619	4828771	R	S	R	S	R	S	S	R	S	S	R	1	0	0	0	0	0	0	1	1	0	0	0	0	0	1	0	0	0	0	0	0	0	0	0	0	0	0	1	0	0
SewageB-088	B1	1,267,278	191,358,978	37	75	571106	4845077	S	S	S	S	S	S	S	S	S	S	S	0	0	0	0	0	0	0	0	0	0	0	0	0	0	0	0	0	0	0	0	0	0	0	0	0	0	0	0	0	0
SewageB-089	B1	1,311,756	198,075,156	39	76	674021	4788969	S	S	S	S	S	S	S	S	S	S	S	0	0	0	0	0	0	0	0	0	0	0	0	0	0	0	0	0	0	0	0	0	0	0	0	0	0	0	0	0	0
SewageB-090	D	1,259,260	190,148,260	35	100	544551	5169268	S	S	S	S	S	S	S	S	S	S	S	0	0	0	0	0	0	0	0	0	0	0	0	0	0	0	0	0	0	0	0	0	0	0	0	0	0	0	0	0	0
SewageB-091	A	1,421,930	214,711,430	42	100	546123	4905176	S	S	S	S	S	S	S	S	S	S	S	0	0	0	0	0	0	0	0	0	0	0	0	0	0	0	0	0	0	0	0	0	0	0	0	0	0	0	0	0	0
SewageB-092	B2	1,256,232	189,691,032	36	94	719707	5079081	S	S	S	S	S	S	S	S	S	S	S	0	0	0	0	0	0	1	0	0	0	0	0	0	0	0	0	0	0	0	0	0	0	0	0	0	0	0	0	0	0
SewageB-094	F	1,304,080	196,916,080	39	44	722204	4833720	S	S	S	S	S	S	S	S	S	S	S	0	0	0	0	0	0	0	0	0	0	0	0	0	0	0	0	0	0	0	0	0	0	0	0	0	0	0	0	0	0
SewageB-095	B1	1,315,718	198,673,418	38	88	632738	4832230	R	S	S	R	R	S	S	R	S	S	R	1	0	0	0	0	0	0	1	1	0	0	0	0	0	1	0	0	0	0	0	0	0	0	0	0	0	0	1	0	0
SewageB-096	B2	1,416,714	213,923,814	42	43	928555	4893833	S	S	S	S	S	S	S	S	S	S	S	0	0	0	0	0	0	0	0	0	0	0	0	0	0	0	0	0	0	0	0	0	0	0	0	0	0	0	0	0	0
SewageB-097	A	1,542,266	232,882,166	47	66	1154442	4757700	S	S	S	S	S	S	S	S	S	S	S	0	0	0	0	0	0	0	0	0	0	0	0	0	0	0	0	0	0	0	0	0	0	0	0	0	0	0	0	0	0
SewageB-100	B2	1,239,938	187,230,638	36	52	509004	4890572	S	S	S	S	S	S	S	S	S	S	S	0	0	0	0	0	0	0	0	0	0	0	0	0	0	0	0	0	0	0	0	0	0	0	0	0	0	0	0	0	0

Supplemental table S2. Strain KAEDA-061 specific non-synomious mutations in comparison with strains KAEDA-068 and KAEDA-073.

scafold	scaffold length	SNP position	base in KAEDA-061	base in KAEDA-068 and KAEDA-073	gene product	mutation	gene start	gene end
scaffold141	130211	120222	T	A	potassium transporter Kup	T97S	119931	121798
scaffold161	274020	52361	T	C	ATP phosphoribosyltransferase HisG	Q206*	51743	52641
scaffold37	138260	20852	A	C	dihydropteridine reductase NfsB	V187F	20760	21412
scaffold48	648066	524690	A	T	nitrate/nitrite transporter NarU	S295C	524337	525572
scaffold7	432702	163937	G	A	LPS assembly outer membrane protein LptD	K131E	163547	165901

Supplemental table S3. Strain KAEDA-061 specific genes in comparison with strains KAEDA-068 and KAEDA-073.

gene	product	sequence
KAEDA-061_specific_gene_01	shufflon system of plasmid conjugative transfer	ATGAAAAAATATGACCGAGGCTGGGCATCGCTGGAAACTGGCGCGGCTTTACTGATTGTT<br>ATGCTCCTGATTGCCTGGGGAGCGGGCATATGGCAGGACTATATTCAAACAAAAGGATGG<br>CAAACTGAAGCACGTCTTGTCAGCAACTGGACCAGTGCGGCCCGCTCTTATATAGGGAAA<br>AACTACACGACTCTCCAGGGCAGTAGTACCACAACAACTCCTGCCGTTATCACGACAACC<br>ATGCTGAAAAATACCGGCTTTTTGTCCAGCGGGTTTACTGAGACAAACAGCGAGGGGCAG<br>CGGTTACAGGCATATGTGGTTCGAAACGCCCAAAACCCGGAATTACTACAGGCAATGGTT<br>GTATCCAGTGGTGGCACACCTTATCCAGTGAAAGCACTTATCCAGATGGCTAAGGATATT<br>ACCACTGGTCTTGGTGGATATATCCAGGACGGCAAAACAGCCACAGGTGCATTACGTTCC<br>TGGTCAGTAGCTTTAAGTAATTATGGTGCCAAAAGCGGTAACGGGCATATTGCCGTATTG<br>TTATCGACAGATGAACTTAGTGGTGCAGCTGAGGACACTGATCGTCTTTACAGATTCCAG<br>GTCAATGGTCGCCCTGACTTAAACAAAATGCACACGGCCATTGATATGGGATCAAATAAC<br>CTGAATAACGTTGGGGCAGTAAATGCCCAGACAGGTAATTTCAGCGGCAATGTGAATGGT<br>GTAAATGGCACTTTCAGCGGTCAGGTAAAAGGCAATAGCGGAAACTTTGACGTAAATGTC<br>ACCGCTGGCGGTGATATCAGAAGTAATAATGGTTGGTTAATTACTCGTAACAGTAAAGGC<br>TGGCTCAATGAAACTCACGGTGGCGGATTTTATATGTCCGATGGATCATGGGTTCGAAGT<br>GTAAACAACAAGGGCATCTATACCGGCGGTCAGGTGAAAGGCGGTACTGTTCGGGCTGAT<br>GGTCGCCTTTATACTGGTGAATACTTACAACTGGAAAGAACTGCCGTTGCTGGCGCATCA<br>TGTTCGCCTAACGGCCTTGTAGGCCGCGATAATACAGGGGCAATACTTTCGTGCCAATCC<br>GGTACGTGGGGGACAATAGGTGGAAAACTCAAAGTTACTCAGCTTTCCACCACAGGTTAT<br>CTGGGGCAATTCGACTTCTGTGCCATTGCCAGAATGGGCAACGCAGAGGATGCCCACTAC<br>TGCCAGGTAGTTGAGAGCCCAGCAGGTTCACGCAAATGGTACAAATACGAGCATAAGACA<br>GGTTGTATTGCGTCGTGTGTAACGCTCAATTAA
KAEDA-061_specific_gene_02	shufflon system of plasmid conjugative transfer	ATGCTAACCTCAAAAGAGGCAAAAAATAATGCCAACAATCATTCTCTGCAAGCGTGCCAA<br>TCCGGTACGTGGAAGACTTCTGGTTCGCTCAATGGTTCTTACACAAACTTAGGTTCACAT<br>AGAGGTTCATTCTCAGGGCGGAATTCAGGGGGCAGTACATTGTTTATTTATGCATCTGGA<br>GGTAATGGAGGATCTGCCGGAGGTGCATGTGCAAATACATCCCGACTGCAGGGATATGTT<br>GGTGGGACGCTAATTAGCGTGAATGCCAGCAATAACCCTGCATATGGAAAAACAGCCTTT<br>ATCAGCTTTGCTGTACCTGCAGGTACTTCCTATCAGATAACATCCTATCCAACAGAAAAT<br>ACATCATGTGGTGCCGGGGTATTTTCAGTATTTGGATATCAAACTTAA
KAEDA-061_specific_gene_03	hypothetical protein	ATGGATAAAGATACTGTTTTAAAAATGTTGAATCCGGCTTGTTTAAAAAAAGCGGATGAA<br>TGGGAATCTCCTACATGTGGAGAAGTTCGGGCAGTCATTGGCCTGACCGGAATGAGCGGC<br>TCGCAGTTGGCCAAAAAACTTGGCTTAAAAGATAGTCGCAATGTTCGCAACTGGCAGATG<br>TTAAAGGAGTCCACATCCAGATCCAGCATTCCGTATGCAGCCTGGGCGCTGCTGTGTTAC<br>TTCGCTGGGTTAGGGTTAATCTTCATTGATAACAAAACAGATGTTTGA
KAEDA-061_specific_gene_04	hypothetical protein	ATGAAAAATATAACTGTTTCTGATTTTGACTCCAAAAATACACTTGTCGATAATTTTAAA<br>TTATCGGGGTTTGATGTCGTTGTTACTGATGTAAAAGGAGACAGGGTCAGGATTGTTGAT<br>GCTTTACCTGAACTTCTTCTTGGAAACCTTAAGCTTAAAAATGCTGATGGTCATAATATT<br>GATCCAACGGAAATTGTCTCCTCTATAGACGCATCTAAACTCGGGTTAGATGTGGCTGTT<br>CTTGGGGGGCTAATTGATGGTGCTGATATGAAAGGCTCCTCCCAAAAGGCACAATCGGAT<br>AAGAAAGAAGATGATAATAAAAGACTCACAGAATTACTTAACGAAAATAAAAAACTAAAG<br>GAAGAGCTTGCAGACGCACAGCAACCAAAAGAGTCACCAAATAAAATCGAGGTGATAGCG<br>GAAAAGACAGAAACCATCATGCAACAATATCAGGAACCACCTCTGGAAGAAAGTGTTCCG<br>AAAATTTATACCCCGAAGAAACCTCAGTTGATTTTAGATGATGCTGGCGGATCATCTGTT<br>TCCAGTTCTCAGGGGGCAAATCCTTCCAGGCAGGAGGGAAGTGATGTAACTGGAAATGCA<br>TCTACTACTTTGAACGATGTCATACCTTAG
KAEDA-061_specific_gene_05	hypothetical protein	ATGATGCTCGGCTGGATGATTGCATTTTTAGCGGTTGGTTTTTTAATCGGTATTGTGGTG<br>ATGTCCAGTTGCATCAATGACTACATTAAAAGCGGTGTTATAGAACGACGCGGTCGTATT<br>TATCGCATTGTAGAAATAACCCACACAGTGAAGGAGATTAAGGATGATCGTATTAAGTAA
KAEDA-061_specific_gene_06	phage antitermination proitein Q	ATGAATAACCAGTATTTACAGTTTGTGCGTGAGCAGCTCATTATCGCCACCGCTGATTTG<br>AGTGGGGCAACAAAAGGTCAGCTTGAAGCCTGGCAAGAGAATGCCATGTTCGATACAGGG<br>CGTTACAGGCGAAAAAAAATCCGGTACCGCGATGAAGTGACTGGAAAAATGATAACGCTG<br>GATAATCCACCAATTCCGGGAAAGCAATCGCTGGCGAAGGGGACGTCAATTCCTCTGGTC<br>AGTCCGGTTGAGTTTTCGACATCATCGTGGCGGCGGGCTGTTCTGTCTCTTGAAGAACAT<br>CATAAAGCCTGGTTGTTGTGGTGTTACAGCGGGAGTATTTGTTGGGAATATCAGATCGCG<br>ATAACACAGTGGGCGTGGAATGAATTTAATACTCAATCCGGTACCAGAAAAATTGCAGGG<br>AAAACGCAGGAACGCCTGAAAAAATTAATCTGGCTGGCGGCGCAGGCAGTAAAAGCAGAA<br>CTTTTTGGTGGGGAAGGTTATGAATACCAGGAGCTGGCATTACTGGCGGGAGTGACAACT<br>AAAAACTGGTCCAAAACATTTACTGGTCACTGGGTTGCAATGAAACACACTTTTCATCGG<br>CTGGATAGTGAGGCTTTATTGTTCGTGACGAGAACACGTTCAAAACAAAAGGCGGCATTT<br>TCACTGTAA
KAEDA-061_specific_gene_07	AraC family transcriptional regulator	GTGGGTGATTTTATCCAACCGATGTTGCTGGGCATTAGCTTTATCAGGTTGGCAGCGTTT<br>GCAGGGGCGAAATCCGGCGGCGAGTGCCTCGCTGGCATTTGCGTAGAAGGAGACGTTTTC<br>CCGCAAAGCATGTCTGGCGCGGCAAGACGGACGGCAAAAGATGCCTGTGGTGCGCACGGC<br>GAAAACGAATTCGCCGTCGGCATTCGGGTCGCGGGCTAA
KAEDA-061_specific_gene_08	type VI secretion system-associated protein	ATGGCCTGTAACGTGGCTGTCGCATCCGGCGGGGTCGTGGTGGCGACGGCGACCGCCACA<br>CCGGCCATTGACGATGCTTCTGCGGGCGCGGCAGTGGCGGGTAAGCCCGTCATCAGGCCC<br>GCGATAAACCATCCTGTCTGCCGGAAAACGCGCAACCTGCTCATAAAACCGTTTCATCCT<br>TTGTGA
KAEDA-061_specific_gene_09	partitioning protein ParA	ATGACTATCTCTGTAGGTATTTTCAACGGAAAAGGCGGCGTAACCAAATCAACAATTGCC<br>CGTGCTTTAGCAGTTGCTTATGCAAAGGCTGGCTGGAATGTATGCCTGATCGATATGGAT<br>GTGCTTAACGGAACGGTAACGCGTTGGGTACAACGCAGAATGGCCAACAATATCGAACCA<br>GCAATCGCGGCGCAGCCGTGCGGCACACCATCACAAGTCCAGAAAATCATCGAATCAGAA<br>GTATACGACCTGGTTATCGTCGATGGCGGTGCCTACGCATCTGAATCAGTACCGAAGCTC<br>TCTGAATTTCTGGATATGGTTATTTTGCCAACCAGATTCTCTACCGATGACCTGGAAACC<br>ACCGTTAACACTGCTCACGGCATCGTTAAAAAGGGTGTACCAATCAAGAAAATTTGCATG<br>GTGTTTTCCGGCGCGGCTGAAAACGAAGCTGACTATATTGAAGCCCAAGAGTACCTGGCT<br>AATACGCCATACTTTGTTGTTGATAAGTACATTCCCCACAAGCCAGCTCTGAGCAAAGCT<br>CAGGATAAGGGCTTAAGCCTCATTGAGTGCTCATACGTTGCGCCACGCAAAAAGGCAGAC<br>GACGTTATTCAGGGGATTATCAACCAGCTTGAGGCTCTGACTACTGCCGCGTAA
KAEDA-061_specific_gene_10	hypothetical protein	ATGAAACGCAAAATTATTAACCCCCAAGATGTGATTAATTTCCTGACCGATCACCCATGT<br>TCAAAAGTGGACGTAATCGCGGAGCACATTGGTGTGTCGCAGAACTCTATGCGTTGCAGA<br>CTGAGAAGCATGGTTGCCGAAGGACAGATAACAACCAGGAAAATAAATGGTTGCCTGTAC<br>TACTCCGCTAAACCGGAATTGCCATTTGGCATGAATCCTAACTCCATGTTGTTTAACACC<br>TTATTAGCCAAAGTTAAGCCGTTGAGAGAGGCTGTGGCATGA
KAEDA-061_specific_gene_11	hypothetical protein	GTGGCTGACAGAATTGAGATAAAGATGGATTTTTCTTCTCAGGATATTCAACGACAACTC<br>CAGCGCCTTGAAGAACGCGAATTGCCGTTCGCAATGGCGCTTGCGGCAACCAGAACGGCA<br>AAGGCTTCTCAGGCTGCGATTAAGAATGAAATTAATCGGGTATTTGACAGGCCCACGCCG<br>TGGATTCAGAACTCTACTTACGTTTTGGCCGCAAAGAAGAGTGATCCTACAGCCATTGTG<br>TATGCTCGTGAATGGGGAGGAACGCCAGCCCCTGTAACGTTGACTCCGCAAATCGAAGGT<br>GGCCAACGACAGTACAAACGCTCAGAAGGGGCGCTAAGGGCTGGTGGATATTTGCCTAAC<br>GGCTGGCAGCTTGCTCCTGGTCCCGGCGCAAAGCTGGACAAATACGGGAATATTTCGCGG<br>GGGCAATTACAGCAGGTCTTATCTGGCCTAAGAGTTCAGCGAGATGCGCATCAAAATCGC<br>CGTCAGGGCAAGCCTACGGAGTTTTTTGTTGTTCGACCTGGTACAAGTAACCCGCTTCAA<br>CCAGGCGTCTGGCAGCGCGTTGGGCGGCGTCCTACGTTGATCCTTACGTTTATCCAGCAA<br>CCTAACTATTCGCAGCGTCTTGATTGGCATGGCGTGGCGCTGCGTGCTGGTGAGGATGCG<br>TTTGCTGATGAAGTCACAAAGGCTATTGATGACATTCTTTCTAAGACCTTCTCTCGTTAA

KAEDA-061_specific_gene_12	phosphoribosyltransferase	GTGATCCAGGCGCGTGAATCAAAATACATCATGATGCACGCACCGACCGAACGTCTGGAT<br>GAAGTCATCGCCCTGCTGCCAGGTGCCGAACGCCCAACCATTCTGCCACTGGCGGGTGAC<br>CAACAACGCGTAGCGATGCACATGGTCAGTAGCGAAACCCTGTTCTGGGAAACGATGGAA<br>AAACTGAAAGCGCTGGGTGCCAGTTCAATCCTGGTCCTGCCGATTGAGAAGATGATGGAG<br>TGA
KAEDA-061_specific_gene_13	hypothetical protein	GTGAGCAAGAAAAATGCCGGATGGGTCAGGCCAATGAAGGCGAATAAACATCATTATTTT<br>AATGAAGGTGAAGTAACCAGCATTTGCGGCCGTTGGATGTATTTTGGTGATTACCGTGAG<br>CCAGACACTTTTGAGAGTCCTGATGATTGCGTGTCTTGTCGCCGGAAATTGAACAAGGTG<br>GCTTCTAAATGA
KAEDA-061_specific_gene_14	transposase	GTGAGTAAGAAGAAAAACACCACAACGCCCACGCCGCATGATGCCGCGTTCCGGTCGTTC<br>CTGGCGAATCCCGACGTCGCCAGAGATTTTCTGGAACTGCATCTTCCGGCGGAGTACCGG<br>CAGTTGTGCGACCTGTCCACGCTGAAGCTGGAACCCGCCACCTTTGTTGAGCCGGACCTG<br>CATCAGTACGCCAGCGATATCCTCTGGAGCGTGAAAACCACCGGGGGTGAAGATGGCTAT<br>GTTTATACGCTCATCGAGCACCAGAGCACCGAAAATCTGTACATGCCTTTCCGCATGTTA<br>CGTTACAGTGTGGCNNNNNNNNNNNNNNNNNNNNNNNNNNNNTACTGTTCTATCATGGTGAG<br>CGCAGCCCGTACCCGTACAGCATGAACTGGCTGGACTGTTTTGAGAATCCGGCACTTGCG<br>GCTAAAATATACACAAAGCCGTTTCCGCTGGTTGATATCACTGTCGTTGATGACAATGAA<br>ATCATGAACCATCGCCGGATGGCCGCACTGACGCTGCTGATGAAGCATATCCGCCATCGT<br>GACATGATGGAGCTGCTGGACAAACTCCCGCAGGTCATGGTGGAAATTTCAGATGAGCAG<br>GTGCGTGTTCTGATTCATTACATCGTTAACGCAGGGGACTCTGTATCACCGGAATTTATG<br>CGGGCGCTGGCTGAGCGTCTGCCGCAGCATGAGGATAAACTGATGACTATCGCTGAACGT<br>CTTGAGCAAAAAGGTCGCCAGGAAGGCAGGATGGAAGGACGCATGGAAGGAGCGCTTGAA<br>AAAGCCTTGGCTATTGCGTGCCAGCTTCAGAAAATGGGGATGACGCCGGAGCAGATTAAG<br>CAGGCTACCGGACTTTCCGATGACGAACTGAAGAAAATCACTCACTGA
KAEDA-061_specific_gene_15	phage tail protein	ATGACTAAAAATATCCGTAATCTGGCACTGGCAACGATGTCGGGGTTTCGCCATAAAACC<br>GTTGATGTGCCTGAATGGGAGGGAGCAACGGTTGTGTTACGGGAACCTTCTGCAGAAGCC<br>TGGTTGCGCTGGCAGGAGATCGTTAGAGCAAAAGATGATGAGACACCGTTATCCGTAGCG<br>GAGCGCGCCCGCCGAAATCTGGAGGCAGATGTTGAACTGTTCATTGATGTTCTGTGTGAT<br>ACCGGACTGCAACCTGTATTTTCAGAGGATGATCGTGAACAGGTGATTGCCGTGTATGGC<br>CCGGTGCATGCGCGGCTTCTTCGGCAGTCTCTGGAACTGATCAGTGATGCCGGCGAGGTT<br>AAAAAAAAGTAG
KAEDA-061_specific_gene_16	phage portal protein	ATGAATATTCTTGATCGAGTTATTGCGCCGTTTTCACCTCAGAGGGCATTAAATCGAGCT<br>TTGGCAAGAAAACGGTTAGAAGCACTTGAAGGTATTAATAACCTTGGATATTCACGTCAT<br>GGTGCAAGCACTCATAAAAAATCATTAAGGGGTTGGTTTAGTAAAGCTGGTTCTCCTGAT<br>GATGATATTGTTAAAAATATAGATAAATTGCGTGAGCGTTCTCGTGATCTCTTCATGGGA<br>AACCCGCTATCGGTTGGCGCAATAAAAACTATCAGGACAAACGTTGTTGGTTCCGGTTTA<br>AAACTAAATGCGAACATTGATGCCGATTTCTTGGGGATGACGCAGGAAGAAGCGCGAGCA<br>TGGGAAAAGCATGTTGAGCGTGAGTTTAGATTATGGGCTGATTCTCCAAATTGTGATGCA<br>TCAAGAATGTGTACTTTTGGACAATTGCAATCATTGGTCCAAATATCTGCGTTAACATCT<br>GGCGATATATTTGCTGCGCTACCAGTTATTAAGCGTAAGGGTGTTATATATGATTTGTGC<br>GTCTATTTAATTGAAGGTGATCGTGTTTGCAATCCAGAGGATCGTTTTATACCAAATCTT<br>TACGGTGGCGTAGAGGTTGGTGAATTTAGCGAGCCTGTGGCGTACTGGGTTGCAAAACAT<br>CACCCAGCCGGGACTTCTGGTTTTGTAACCCGCAAGTGGGAACGTATTCCCGCATATGGG<br>AGTAAAACAGGAAGAAGAAATATTCTGCATGTCATGCAGGATTTTGAAAGGCCTGGGCAA<br>CGCCGTGGCGTTCCTGTCCTTGCTCCTGTTATTGAAGCATTGAAGCAGTTAGGACGATAC<br>ACCGATGCTGAATTGGTTGCTGCGGTTGTGTCAGGGATGTTTACTGTCTTTATTAAAACA<br>GACGCTCCTGATGGACCAGTCGGTGAATCGGGTATCCCTCAGTACGAGCAGATCGATAAC<br>CATGATGATAACACCCAAGAAATGGGGAATGGCTCCATAGTAAGTTTGGGTGAAGGTGAG<br>TCGATAGATACAGCAAATCCCGGGCGACCAAATACGGCATTTGATGGTTTTGTTGTCGCC<br>ATATGCCGTCAAATTGGGGCTGCTTTAGAACTCCCTTACGAACTCTTGGTAAAGCATTTT<br>ACAGCAAGTTATAGCGCAAGCAGGGCCGCATTACTTGAAGCCTGGAAAATGTTCCGTATG<br>CGACGTGACTGGATGGTTCAGTCATTTTGCCAACCGATCTATGAGGAATGGTTGGCCGAA<br>GCCGTTGCTAAAGGTCGTGTTATTGCCCCTGGTTTTTTTATGGGCCTGAATATCGCGCT<br>GCATGGAGTGGCGCTCAATGGTATGGCCCATCACAAGGCCAATTAGATCCCCTTAAAGAA<br>GTCAAAGCTGCAAAACTGCGTGTTGAGGAAACATTCTCTACCCGTGAAAAGGAAGCGGCT<br>GAAATGTCAGGTCTTAACTGGGAAGAAACCGCACAAATTTGTGGCAGAGAAGAGAATGCT<br>CGCCGTGAGTTGGGGCTGATTACGCCTCCTGTTTCTGAGGTAAATGAACAAAATATGGAG<br>ACAGATGATGCCTAA
KAEDA-061_specific_gene_17	hypothetical protein	ATGATCGTATTAAGTAAGCGGGAGAAGGAAACGCTTCATGAAATCAGTAAGTGGTCAGAG<br>TTCCCTGAGTACTGGAAGCCTAAAACGCGGGCTAAGTTAGAGCGGTTAGGGTTGGTTGCA<br>AACGTTTCTGAAACGAAGTGTTCGGCCAACTACCAGTTAACTGATAAAGGGAAAGTATTG<br>CTACAGCAATTAGTAGAATCAGGAGTGTTAAAATGA
KAEDA-061_specific_gene_18	hypothetical protein	ATGAATAAACAAGTAATTATTTCTGACTGGATCAACAACCCTAACAGCCTGTTAAGCACT<br>GATACTGGCTACCTATTACGCCATGTTAATGGTCGTATGGTCAAAAGCGATGAGCACGAA<br>ACCTTTTTCTTTGTTGAAGACGATGGGCGCATCTATGAGGATGGTTACTCTTATGAAGCC<br>CAAACTGGTTGTATCCCTGCGGAGCTGGTGGACGTAACAGAAGATCTGCGCAAAGCATGG<br>CTTGAACAGCAGCAGCGCGGAGATGATTACGTAAACACCTATCAGGAACGCCAGAACGCG<br>CGTCTTGCCCGATATATTGCTCGTGCAGAAAAAGCCAGAAAAGAAGGTGCGGTAGCGCAT<br>AAGCGAGCGCATGACCTGTTGGATGTGATCCCGTTAGGCCAGCCGATTTTAGTTGACCAT<br>TATAGCGCGAAAGGCCATCGCCGCCGTTTGTCTAAAGCTGATGCGTTATTCAGAAAAGCG<br>TTTGTTGAATGCGAAAGTAAAGCATCACACTACGAGAGCAAAGCCGCAGGTGTTGGTCGT<br>AATGGAATTTCGTCTGATGATCCTGATGCGCTATTTAAGTTGCTTCGTAAGCTGCAAGGC<br>TGCATGAAGTCACACGTCAAAATGAAGGCCGCAAATAAGGCCATTCGCAAATACAAAAAA<br>GACCAATTACAGCAGCTTTCTGCATTAATCGATCTCAGGTTTACAGAAAGCGAAGCCAAA<br>GAGTTACTGGCCGGAGATTTTTGTGGGCGCATCGGATTCCCTTCATACGCACTGAGCAAC<br>AATAACGCCGAGATCAAACGACTACAGAGCCGTATTAAGGAGCTTGAATCGGTCAAGTCT<br>GTAACAGGAGCACAGCGCGAAGAATACGACGACTTTTCTATGGAGATAGATCCAGAGGAT<br>AACCGCATCCTGTTTTATTTCCCTGGCAAACCAGAGGCAAACATTCGTTCGCTGCTTAAA<br>TCACGCGCCTTTAAGTGGAGTCCAACCCGTAATGCATGGGTTCGCAAGATTACTCCAAAC<br>GCCCTGGCTGATGCCCGATATCTAAAAGAATCGCTATTGAAAGCCTGA
KAEDA-061_specific_gene_19	hypothetical protein	ATGACAATCACCACCATAATAACTCCCCAGTTCCGGATGATGGCGGTGATACGCCTGTTC<br>CGCCAGACGACGGCGGTGATACGCCGGTTCCGCCAGATGACGGCGGTGATACGCCGGTTC<br>CGCCAGATGACGGCGGTGATACGCCAGTTCCGCCAGATGACGGCGGTGATACGCCGGTTC<br>CGCCAGATGACGGCGGTGATACGCCGGTTCCGCCAGATGACGGCGGTGATACGCCAGTTC<br>CGCCAGATGACGGCGGTGATACGCCGGTTCCACCAGATGACGGCGGTGATACGCCGGTTC<br>CACCAGATGATGGCGGTGATACGCCAGTTCCAGACGAAGGCGGTGATACGCCTGTTCCGC<br>CAGACGATGGTGGCGATACCCCGGTTAAACACGATCCTGTCGTCTATAAAAATGACGTAA
KAEDA-061_specific_gene_20	hypothetical protein	ATGATTCCATACATCTCATTAGCTTTTATGAGTGGCTTCCTTATCGGCTTCGGCATCTGT<br>CGTGATTTAATTAAGCAGGAACTTAAAACCAAAACACTGTGCATCGGAAAGCGTGTGTAT<br>CGGGTAGTTCATGAAACAAAGGTTAGAAAATGA
